# *TNFRSF13B* polymorphisms counter microbial adaptation to enteric IgA

**DOI:** 10.1172/jci.insight.148208

**Published:** 2021-07-22

**Authors:** Jeffrey L. Platt, Mayara Garcia de Mattos Barbosa, Daniel Huynh, Adam R. Lefferts, Juhi Katta, Cyra Kharas, Peter Freddolino, Christine M. Bassis, Christiane Wobus, Raif Geha, Richard Bram, Gabriel Nunez, Nobuhiko Kamada, Marilia Cascalho

**Affiliations:** 1 Department of Microbiology and Immunology and Department of Surgery,; 2 Department of Surgery,; 3 Department of Biological Chemistry and Department of Computational Medicine and Bioinformatics,; 4 Department of Medicine, and; 5 Department of Microbiology and Immunology, University of Michigan, Ann Arbor, Michigan, USA.; 6 Department of Pediatrics, Boston Children’s Hospital, Boston, Massachusetts, USA.; 7 Departments of Oncology, Pediatric Hematology/Oncology, and Pediatrics, Mayo Clinic, Rochester, Minnesota, USA.; 8 Department of Pathology, University of Michigan, Ann Arbor, Michigan, USA.

**Keywords:** Immunology, Microbiology, Bacterial infections, Genetic variation, Immunoglobulins

## Abstract

*TNFRSF13B* encodes the transmembrane activator and CAML interactor (TACI) receptor, which drives plasma cell differentiation. Although *TNFRSF13B* supports host defense, dominant-negative *TNFRSF13B* alleles are common in humans and other species and only rarely associate with disease. We reasoned that the high frequency of disruptive *TNFRSF13B* alleles reflects balancing selection, the loss of function conferring advantage in some settings. Testing that concept, we investigated how a common human dominant-negative variant, TNFRSF13B A181E, imparts resistance to enteric pathogens. Mice engineered to express mono- or biallelic A144E variants of *tnrsf13B*, corresponding to A181E, exhibited a striking resistance to pathogenicity and transmission of *Citrobacter rodentium*, a murine pathogen that models enterohemorrhagic *Escherichia* *coli*, and resistance was principally owed to natural IgA deficiency in the intestine. In WT mice with gut IgA and in mutant mice reconstituted with enteric IgA obtained from WT mice, IgA induces *LEE* expression of encoded virulence genes, which confer pathogenicity and transmission. Taken together, our results show that *C*. *rodentium* and most likely other enteric organisms appropriated binding of otherwise protective antibodies to signal induction of the virulence program. Additionally, the high prevalence of *TNFRSF13B* dominant-negative variants reflects balancing selection.

## Graphical abstract

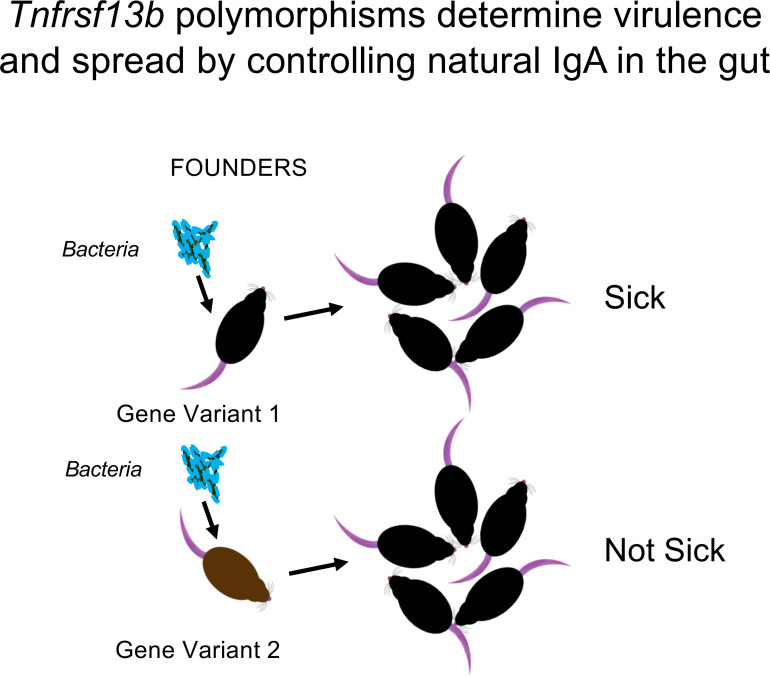

## Introduction

*TNFRSF13B* (in humans or *Tnfrsf13b* in mice) encodes the transmembrane activator and CAML interactor (TACI), a member of the TNF receptor superfamily and has been considered vital to immune fitness. TACI is the receptor for the B cell–activating factor (BAFF) and a proliferation-induced ligand (APRIL). Binding of BAFF or APRIL to TACI activates BLIMP-1 (1), the transcription factor that governs differentiation of B lymphocytes into plasma cells (2–4). Common variable immunodeficiency and IgA deficiency in humans and mice have been associated with *TNFRSF13B* null and dominant-negative mutations (5, 6).

Since *TNFRSF13B* in humans supports a key facet of immune fitness, it may be surprising that surveys of normal populations reveal extraordinary polymorphism — 951 *TNFRSF13B* missense and only 383 synonymous mutations (https://useast.ensembl.org/index.html) — and a high frequency of dominant-negative alleles (7). Most individuals with TNFRSF13B variants that disrupt function are healthy (7, 8). However, this paradoxical diversity is not limited to humans, as *TNFRSF13B* polymorphism also occurs in other species. For example, 17 missense, 2 stop gained, and 2 splice variants have been reported in mice (https://useast.ensembl.org/index.html) (9). The mechanism underlying *TNFRSF13B* polymorphism is unknown, although missense alleles appear to have been selectively retained in populations, and the McDonald-Kreitman neutrality index indicates the locus is under strong positive selection. This is in contrast to genes encoding HLA, which are under moderate purifying pressure (10).

The diversification of *TNFRSF13B* across species, the high frequency of dominant-negative variants, and the evidence of positive selection suggested to us that the biological impact of *TNFRSF13B* function is incompletely understood. Therefore, to explore potential functions of *TNFRSF13B* and pressures for diversification, we tested the effect of allele variants embodying the range of functions of *TNFRSF13B* on resistance to and transmission of *Citrobacter rodentium* in mice, which models enterohemorrhagic *Escherichia coli* in humans (11). Here, we investigated whether the high frequency of *TNFRSF13B* polymorphisms and the frequent dominant-negative phenotypes across species could reflect an adaptation to resist common enteric pathogens.

## Results

### Tnfrsf13b controls resistance to C. rodentium.

We first asked whether or to which extent *Tnfrsf13b* mutations modify susceptibility of naive mice to infection with *C*. *rodentium*. Accordingly, the smallest number of *C*. *rodentium* (108) that reliably generates disease (determined by infecting mice with numbers of bacteria varying from 10^10^ to 10^7^ organisms) were given to WT mice (C57BL/6 mice) and to mice of the same background with (a) mono- or biallelic *Tnfrsf13b* mutations encoding mA144E (homologous to the frequent human A181E mutation) and to mice with (b) fully disrupted *Tnfrsf13b* and monitored excretion of viable organisms in stool during the ensuing month. Because gut microbiota potentially influence the virulence of *C*. *rodentium* (12), mutant and WT mice used in these experiments were cohoused for 4 weeks prior to infection to allow admixture of flora.

**Figure 1:**
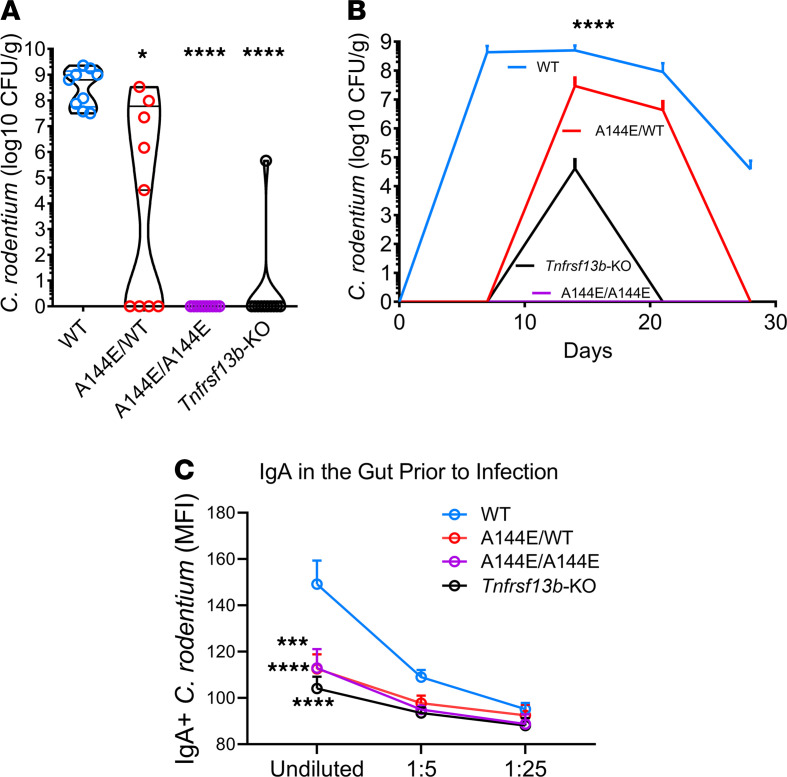
*Tnfrsf13b* mutant mice resist infection with *C. rodentium*. To evaluate baseline resistance to enteric infection with *C*. *rodentium*, WT C57BL/6 mice, and mice with mono- or biallelic mutations in *Tnfrsf13b* (A144E) and mice with targeted disruption of *Tnfrsf13b* (*Tnfrsf13b*-KO) were infected with 10^8^ *C*. *rodentium* by oral gavage, and the numbers of viable organisms in stool were measured by counting CFU/g of feces after 18 hours of incubation on MacConkey plates at the peak of infection. (**A**) Graph depicts the maximum CFU/g feces in the course of infection for each of the infected mouse strains. Values were analyzed by 1-way ANOVA, the Kruskal-Wallis test (*P* < 0.0001), with multiple comparisons comparing values in *Tnfrsf13b* mutant mice to WT showing *P* = 0.0492 for A144E/WT, *P* < 0.0001 for A144E/A144E, and for *Tnfrsf13b*-KO mice. (**B**) Mean number of viable *C*. *rodentium* in stool (expressed in log_10_ CFU/g feces) at various times after infection. Comparisons to C57BL/6 at day 14 yielded *P* < 0.0001 (1-way ANOVA and the Kruskal-Wallis test), and multiple comparisons test to WT yielded *P* = 0.0048 for A144E/WT, *P* < 0.0001 for A144E/A144E, and for *Tnfrsf13b*-KO mice. (**C**) Quantification of *C*. *rodentium*–binding IgA in feces of mice prior to infection. IgA in feces was measured by flow cytometry analysis of IgA^+^ GFP^+^ *C*. *rodentium* and detected with anti-IgA PE-labeled. *y* axis, Average of 3 independent measurements of IgA mean fluorescence intensity (MFI); *x* axis, feces supernatant dilutions. Analysis of results by 1-way ANOVA yielded *P* < 0.0001. Comparisons of undiluted mutant mice antibodies with those obtained from C57BL/6 mice by Dunnett’s multiple comparisons test yielded *P* = 0.0004 for A144E/WT, *P* = 0.0002 for A144E/A144E, and *P* < 0.0001 for *Tnfrsf13b*-KO mice. *P < 0.05; ***P < 0.001; ****P < 0.0001.

After introduction of *C*. *rodentium,* WT mice typically develop a mild diarrheal disease commencing at 5–7 days, reaching greatest severity at 7–10 days, and resolving at 18–28 days; infection typically elicits immunity that imparts enduring resistance to subsequent infection (11). Consistent with that experience, WT mice exhibited maximum excretion of 10^7^–10^9^ viable *C*. *rodentium* between 7 and 21 days after infection and resolution by 35 days. In notable contrast, mice with monoallelic mutations encoding A144E variants excreted on average 10-fold less viable *C*. *rodentium* than WT mice (*P* = 0.0246), and all mice with biallelic mutations and all but 1 with full disruption of Tnfrsf13b excreted no viable organisms (*P* < 0.0001) (Figure 1A). Perhaps more important, the total number of viable *C*. *rodentium* excreted during the course of the experiments by mutant mice was profoundly lower than the total number excreted by WT mice (*P* = 0.0002) (Figure 1B). Thus, *Tnfrsf13b* mutations that disrupt the function of the encoded protein decrease susceptibility to *C*. *rodentium* infection, and this decrease is appreciated days before adaptive immunity might begin to be manifest.

Since *TNFRSF13B* mutations confer baseline resistance to *C*. *rodentium*, we wondered whether resistance could be mediated by natural antibodies, which are known to confer resistance to other bacteria (13, 14). Natural antibodies are thought to be produced independently of pathogen-specific stimulation and thought to be evoked by low-affinity interactions with self-antigens (15). Although *TNFRSF13B* mutations decrease overall production of natural antibodies, including IgA (16), it is possible that natural antibodies specific for *C*. *rodentium* might nonetheless be present and underlie resistance in mutant mice. Moreover, *Tnfrsf13b* mutant mice had less natural IgM but no less natural IgG in the blood (Supplemental Figure 1A; supplemental material available online with this article; https://doi.org/10.1172/jci.insight.148208DS1). However, serum analysis revealed mutant mice had no appreciable natural IgM or natural IgG in blood that could bind *C*. *rodentium* (Supplemental Figure 1B). More important, stool analysis from unmanipulated mutant and WT mice revealed that WT but not mutant mice had natural enteric IgA that bound *C*. *rodentium* (Figure 1C). Thus, the baseline resistance of mutant mice to *C*. *rodentium* infection is not mediated by natural antibodies.

If natural antibodies do not increase baseline resistance to *C*. *rodentium*, perhaps hastened primary antibody responses do so. To detect an early primary Ig response, we assayed the blood and stool of the various strains for presence of *C*. *rodentium–*specific Ig 7 days after infection with that organism (Supplemental Figure 2). A total of 6 of 8 WT and only 2 of 30 *Tnfrsf13b* mutant mice had IgM specific for *C*. *rodentium* in blood 7 days after infection (Supplemental Figure 2A). Both WT and *Tnfrsf13b* mutant mice had *C*. *rodentium*–specific IgG, but the concentrations were low (<10 μg/mL) (Supplemental Figure 2B). Twenty-one days after infection, when adaptive IgG responses are generally detected, all WT, but fewer than one-half of mutant mice, had *C*. *rodentium*–specific IgG. Since antibodies that protect against *C*. *rodentium* target intimin (a virulence factor), we measured the concentration of these antibodies in mouse serum before and after infection. Intimin-specific antibodies were not observed in any strain until 14–21 days after infection and levels in WT mice exceeded levels in mutant mice (*P* < 0.01), (Supplemental Figure 2D). WT mice also produced significantly more intimin-specific IgA than *Tnfrsf13b* mutant mice 21 days after infection (Supplemental Figure 2E). Thus, primary antibody responses in *Tnfrsf13b* mutant mice were not hastened compared with those in WT mice, and therefore differences in early antibody responses in the blood do not explain heightened resistance to *C*. *rodentium*.

### The Tnfrsf13b genotype determines development of virulence by C.

**Figure 2:**
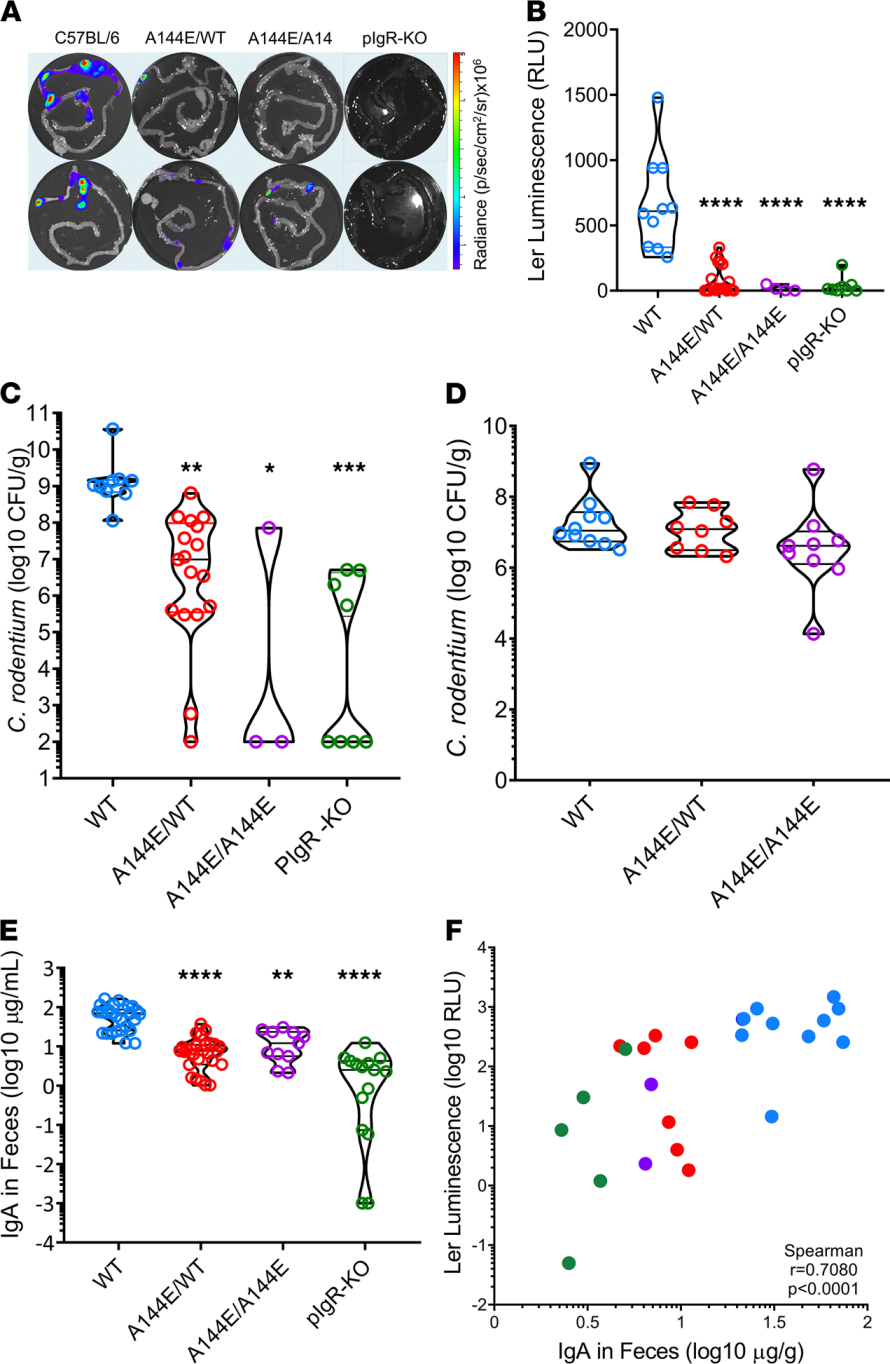
*Tnfrsf13b* mutations block *C. rodentium* virulence gene induction. (**A** and **B**) *Ler* expression measured by bioluminescence of *ler/lux-C*. *rodentium* attached to the intestinal wall of mice infected for 7 days. Shown is *ler* expression in relative light units, RLU, in each strain (*y* axis). RLU reflects the photons/s measured in each image divided by the background luminescence. Comparisons done with 1-way ANOVA yielded *P* < 0.0001; multiple comparisons to C57BL/6 mice (control), yielded for A144E/WT, *P* < 0.0001; A144E/A144E, *P* < 0.0001; and for PIgR-KO, *P* < 0.0001. (**C**) *C*. *rodentium* in feces obtained 7 days after infection for each of the infected mouse strains. Values were analyzed by 1-way ANOVA (*P* < 0.000), followed by the multiple comparisons test comparing *Tnfrsf13b* mutant mice to WT showing *P* < 0.01 for A144E/WT, *P* < 0.05 for A144E/A144E and *P* < 0.0001 for *PIgR-KO* mice. (**D**) *C*. *rodentium* in feces of C57BL/6 or *Tnfrsf13b* mutant mice 14 days following infection with 10^8^ *C*. *rodentium* grown overnight in DMEM medium and expressing *ler*. Statistical analysis was by 1-way ANOVA and the Kruskal-Wallis test followed by a multiple comparisons test. High virulence *C*. *rodentium* infects WT and *Tnfrsf13b* mutant mice equally (*P* > 0.05). (**E**) IgA concentration measured in feces 7 days after infection by ELISA. Comparisons done with 1-way ANOVA and the Kruskal-Wallis test yielded *P* < 0.0001, followed by multiple comparisons test to C57BL/6 mice yielded for A144E/WT, *P* < 0.0001; A144E/A144E, *P* = 0.0049; and for PIgR-KO, *P* < 0.0001. (**F**) Correlation analysis between luminescence reflecting *ler* expression by bacteria attached to the gut walls and IgA concentration in feces supernatants obtained 7 days after infection. Spearman’s test *r* = 0.70894, *P* < 0.0001 indicating that IgA concentration in feces is correlated with *ler* expression. *P < 0.05; **P < 0.01; ***P < 0.001; ****P < 0.0001.

*rodentium.* Since productive infection with *C*. *rodentium* requires acquisition of virulence in the host environment (12), we wondered whether mutations of *Tnfrsf13b* could block such acquisition. To answer that question, we assayed development of virulence by *C*. *rodentium* engineered to express a *ler* bioluminescent reporter gene by fusion of the *ler* promoter with the luxCDABE operon of *Photorhabdus luminescens* (*ler-lux* *C*. *rodentium*) (12). *ler-lux C*. *rodentium* were introduced into *Tnrsf13b* mutant and WT mice, and 5 days later *ler* expression was assayed in the intestinal walls. Figure 2, A and B, shows that *ler* expression was approximately 8- to 40-fold higher in organisms introduced into WT than into mono- or biallelic A144E mutant mice. Decreased expression of *ler* expression in *Tnrsf13b* mutant mice was paralleled by decreased excretion of viable organisms. Thus, mono- and biallelic A144E mutant mice excreted 11-fold and 114-fold fewer viable organisms, respectively, than WT mice (Figure 2C). Thus, A144E heterozygosity and the dominant-negative phenotype are associated with and possibly cause nearly full suppression of *ler* expression and a profound decrease in excretion of viable *C*. *rodentium*. To confirm that concept, we tested whether induction of *ler* before infection vitiate differences between mutant and WT mice. Figure 2D shows that inducing virulence in *C*. *rodentium* prior to infection by culture in DMEM medium at 37°C (17) abrogates resistance of *Tnfrsf13b* mutant mice to that organism. These results demonstrate that resistance to *C*. *rodentium* infection is likely exerted prior to or during acquisition of virulence.

### Natural IgA induces C. rodentium virulence gene expression.

Since *tnfrsf13* governs B cell maturation and production of natural and elicited antibodies, we next determined whether antibodies might exert a previously unrecognized impact on *C*. *rodentium* virulence. *TNFRSF13B* mutations in humans (5, 18, 19) and *Tnfrsf13b* mutations in mice (3, 20) are associated with decreased production of IgA, and IgA maintains homeostasis of commensal bacteria in the gut, which in turn protects against certain gut pathogens (21). However, IgA is not required for the clearance of *C*. *rodentium* (22). Whether natural IgA could influence *C*. *rodentium* virulence has not been explored to our knowledge. To address that possibility, we first measured IgA in feces of unmanipulated *Tnrsf13B* mutant and WT mice. Mono- and biallelic A144E mutant mice had 10- to 100-fold less IgA in feces than WT mice (Figure 2E). IgA in the serum was also decreased by at least 100-fold in mono- and biallelic A144E mutant mice as compared with WT mice (4.0 or 4.7 μg/mL vs. 151 μg/mL, on average, in A144E/WT or A144E/A144E vs. WT mice, respectively). We next determined whether the amount of IgA in feces might influence the development of virulence by *C*. *rodentium*. Seven days after infection with *ler lux* *C*. *rodentium* the amount of IgA per gram of feces correlated significantly with *ler* expression by organisms attached to the intestinal walls (Figure 2, E and F, and Supplemental Figure 3B) and with *C*. *rodentium* CFU (Supplemental Figure 3A). These results were consistent with the possibility that IgA influences the acquisition of virulence by *C*. *rodentium*.

To determine that IgA rather than other properties of *Tnfrsf13b* mutant mice influences the acquisition of virulence by *C*. *rodentium*, we tested whether mice with WT *Tnfrsf13b* but harboring a mutation that compromises secretion of IgA into the small intestine would resist *C*. *rodentium*–like *Tnfrsf13b* mutants. Mice lacking the polymeric Ig receptor (PIgR-KO mice), which transports polymeric IgA from basolateral to apical membrane of intestinal epithelium and hence have little or no IgA in the gut (23), were infected with *ler lux* *C*. *rodentium,* and *ler* expression and *C*. *rodentium* CFU in stool were measured days later. As expected, the PIgR-KO mice had 10- to 10,000-fold less IgA in feces than WT mice at baseline. After infection with *ler lux* *C*. *rodentium*, PIgR-KO mice had on average 20-fold less *ler* luminescence (Figure 2, A and B) and approximately 100-fold less *C*. *rodentium* CFU in feces (Figure 2C and Supplemental Figure 3A) than WT mice. Thus, absence of IgA rather than other facets of the *Tnfrsf13b* mutant phenotype is associated with resistance to *C*. *rodentium* and presence of IgA, with susceptibility to *C*. *rodentium* infection.

**Figure 3:**
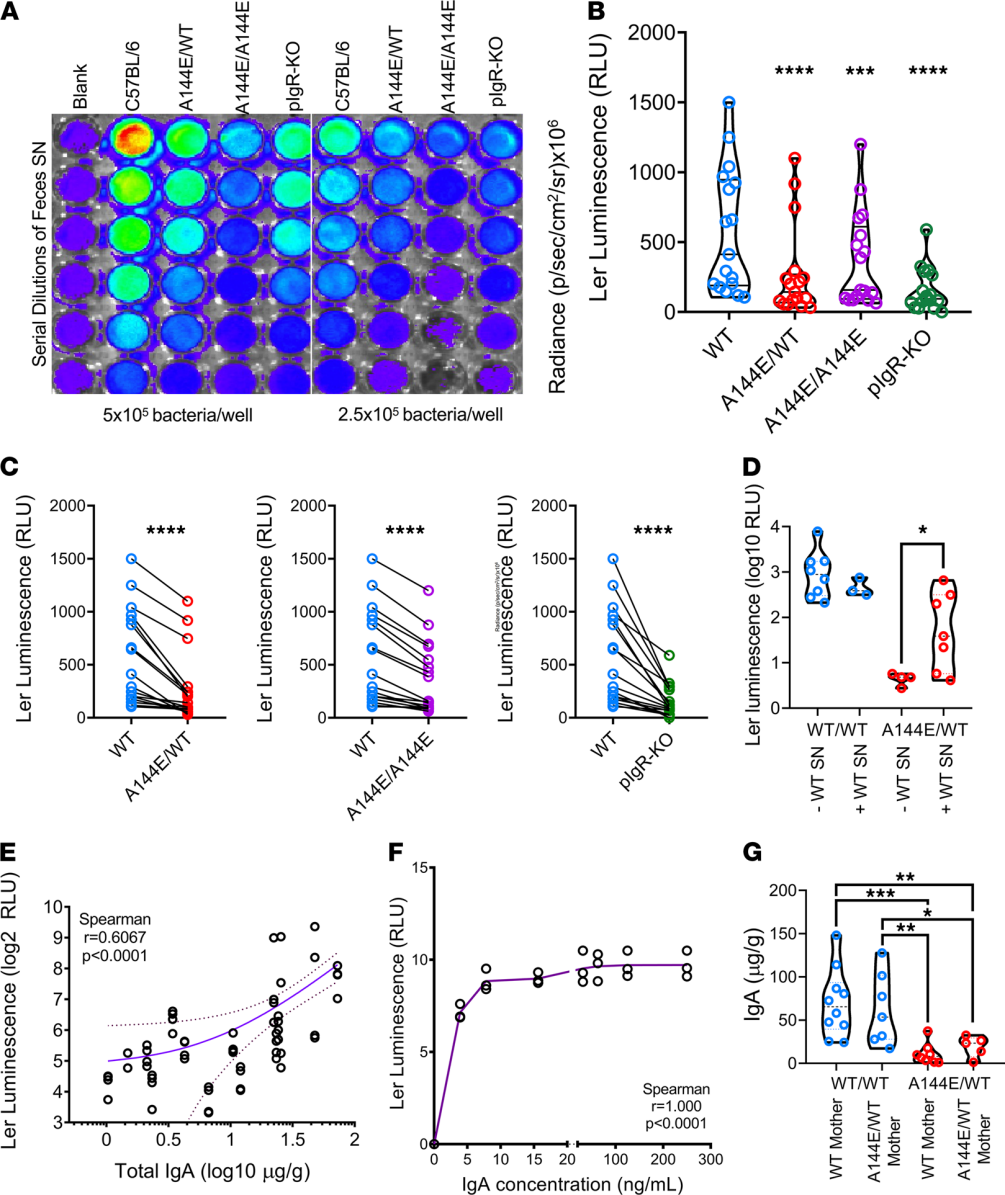
*Ler* expression increases directly with IgA concentration. *Ler* expression measured by bioluminescence imaging (BLI) of *ler/lux C*. *rodentium* incubated with serial dilutions of feces supernatants obtained from noninfected naive C57BL/6, A144E/WT, A144E/A144E, or PIgR-KO mice for 1 hour at 37°C in culture. (**A**) Example of a plate reading in a typical experiment. (**B**) Graph shows *ler* expression detected with BLI and expressed in relative luminescence units (*y* axis), normalized for the feces weight in 12 independent experiments each done in duplicate using 2 different concentrations of *C*. *rodentium,* as indicated in Figure 3A, for each mouse strain (*x* axis). Luminescence data were analyzed by 1-way ANOVA (*P* < 0.0001), and luminescence in each mutant mouse was compared with the luminescence in the C57BL/6 group by the Holm-Sidak’s multiple comparisons test (****P < 0.0001). (**C**) Paired analysis of normalized (to the feces weight) individual measurements of *ler* expression (*y* axis), comparing luminescence obtained with supernatant (SN) from mutant mice with that from C57BL/6 mice, within each experiment (*x* axis). Paired *t* test analysis yielded ****P < 0.0001. (**D**) *Ler* expression measured by bioluminescence of *ler/lux-C*. *rodentium* attached to the intestinal wall of WT or A144E/WT mice infected for 7 days and treated (or not) with feces SN obtained from naive WT mice. Sterile feces SN (200 μL, undiluted and spun to eliminate all bacteria) were administered by gavage twice, 1 day prior and 2 days after infection. The feces SN had, on average, 102.6 μg/g feces of IgA. Gavage of WT SN increased *C*. *rodentium* virulence following infection of A144E/WT mice. Comparisons done with 1-way ANOVA and Kruskal-Wallis test yielded *P = 0.0029 followed by the Dunn’s multiple comparison test, yielding *P* < 0.05 comparing A144E/WT treated or not with WT SN. (**E**) Regression and correlation analysis between IgA concentration in the feces SN (*x* axis) and *ler* expression (*y* axis). Continuous line represents the average and dotted lines the 95% confidence limit. The slope of the curve was different from 0 with *P* < 0.0001. Analysis by the Spearman’s test yielded *r* = 0.6067 and an approximate *P* < 0.0001 (2-tailed). (**F**) Ler-lux *C*. *rodentium* was incubated with serial dilutions of murine IgA in PBS for 1 hour at 37°C. *y* axis, *ler* expression detected with bioluminescence imaging (BLI), *x* axis, IgA concentration in ng/mL. Analysis by the Spearman’s test yielded *r* = 1 and an approximate *P* < 0.0001 (2-tailed). (**G**) IgA concentration in feces of A144E/WT of WT mice born from WT or A144E/WT mothers. One-way ANOVA and the Kruskal-Wallis test yielded = 0.0008 followed by Dunn’s multiple comparisons, indicating *P < 0.05, **P < 0.01, ***P < 0.001.

Given the association of IgA in gut with susceptibility to *C*. *rodentium* infection, we explored whether IgA might in some way promote virulence. Therefore, we next tested whether supernatant of feces containing or lacking IgA induced virulence in *C*. *rodentium*. Serial dilutions of supernatant obtained from fresh feces from naive WT, *Tnfrsf13b* mutant, or PIgR-KO mice were added to *C*. *rodentium* that had been grown in LB medium under conditions that support nonvirulence, and *ler* expression was then assayed. Figure 3, A–C, shows that supernatant from feces of WT mice induced higher (>2.5-fold) *ler* expression than supernatant from feces of *Tnfrsf13b* mutant or from PIgR-KO mice. Further, supernatant from WT feces administered by gavage to *Tnfrsf13b* mutant mice restored virulence (Figure 3D). Moreover, *ler* expression directly correlated with IgA concentration (Figure 3E), consistent with IgA inducing virulence. To test whether IgA (the predominant Ig in stool) or IgG can directly induce virulence, purified IgA (Figure 3E) and IgG (Supplemental Figure 4A) were added to cultures of *ler-lux* *C*. *rodentium,* and luminescence was assayed 1 hour later. Figure 3 and Supplemental Figure 4A show that both IgA and IgG induced *ler* expression by *C*. *rodentium* in a concentration-dependent manner. However, since IgG is not present in the gut early during infection it is unlikely that “natural” IgG contributes to the induction of virulence in a noninflamed gut. However, it is possible that as infection progresses IgG leaked into the gut also contributes to the development of virulence.

The effect of IgA/IgG on *C*. *rodentium* virulence did not depend on complement as heating the supernatants to 56°C for 30 minutes to inactivate complement (24) did not efface induction of *ler* expression (Supplemental Figure 4). Finally, we determined whether virulence-inducing IgA necessarily reflected production by mutant mice or could have been acquired from maternal transfer. IgA assay in stool of various combinations of mutants or WT progeny of mutant or WT mothers revealed the IgA concentration in the gut was determined by the genotype of the progeny and not by the genotypes of the mothers (Figure 3G).

### The Ig genes encoding C.

*rodentium–bound enteric IgA^+^ in C57BL/6 and in A144E tnrsf13B mutant mice have distinct properties*. Our findings suggest that *C*. *rodentium* apparently evolved to co-opt some property of gut IgA to signal induction of the virulence program, and various disruptive mutations of *Tnfrsf13b* in mice and *TNFRSF13B* in humans were sustained possibly to avert infection and transmission of this class of organisms. Since IgG, which is not present to any great extent in normal noninfected gut, and Ig from all WT animals tested induce virulence in *C*. *rodentium*, we reasoned that the “active” region of Ig likely depends on conserved sequences, in the F(ab)_2_ or the FC domains, or possibly on the associated J chains and secretory piece. To determine if F(ab)_2_ sequences were shared, we compared the sequences of IgH and IgL genes from IgA^+^ *C*. *rodentium*–specific B cells isolated from the Peyer’s patches of mice 14 days after infection or 5 days after reinfection. Following reinfection of 10 clones isolated from WT mice, 2 B4 IgC03 and D3 IgC09 were found repeatedly (3 and 2 times, respectively). All of the clones were encoded by 1 of 3 VH regions; VH1, VH3, or VH5. A total of 6 of 10 Heavy chain (HC) sequences were greater than 98% homologous to germline (Supplemental Tables 1 and 2). The HC Ig sequences also had short CDR3 and short N regions [nontemplated nucleotide additions during V(D)J recombination], as would be expected of antibodies arising from fetal lineage precursor cells with restricted terminal deoxynucleotidyl transferase (TdT) activity (25). Only 59% of the mutations observed were nonsynonymous, suggesting absent or weak antigen selection (Supplemental Table 2). These properties are characteristic of natural antibodies (26). In contrast, the IgH sequences from A144E homozygous mice were characteristic of elicited responses. Upon reinfection, only 40% of the clones from mutant mice contained germline sequences and none were repeated, indicating greater diversity than the WT (Supplemental Tables 3 and 4). *C*. *rodentium*–specific IgA clones from A144E homozygous mice had longer N regions than clones from WT mice and although the overall mutation frequency in clones from A144E homozygous mice was low, on average (3.6%), 70% of the mutations were nonsynonymous, suggesting antigen selection. *Tnfrsf13b* mutations do not impair differentiation of IgA^+^ B cells, instead they impair differentiation into plasma cells. Accordingly, *Tnfrsf13b* mutant mice did not have decreased frequency of *C*. *rodentium*^+^ IgA^+^ B cells or of CD19^+^ B cells in Peyer’s patches (Supplemental Figure 5B) compared with WT mice; *Tnfrsf13b* mutant mice had as many T cells and increased frequency of B cells in the spleen compared with WT mice (Supplemental Figure 5C).

**Figure 4:**
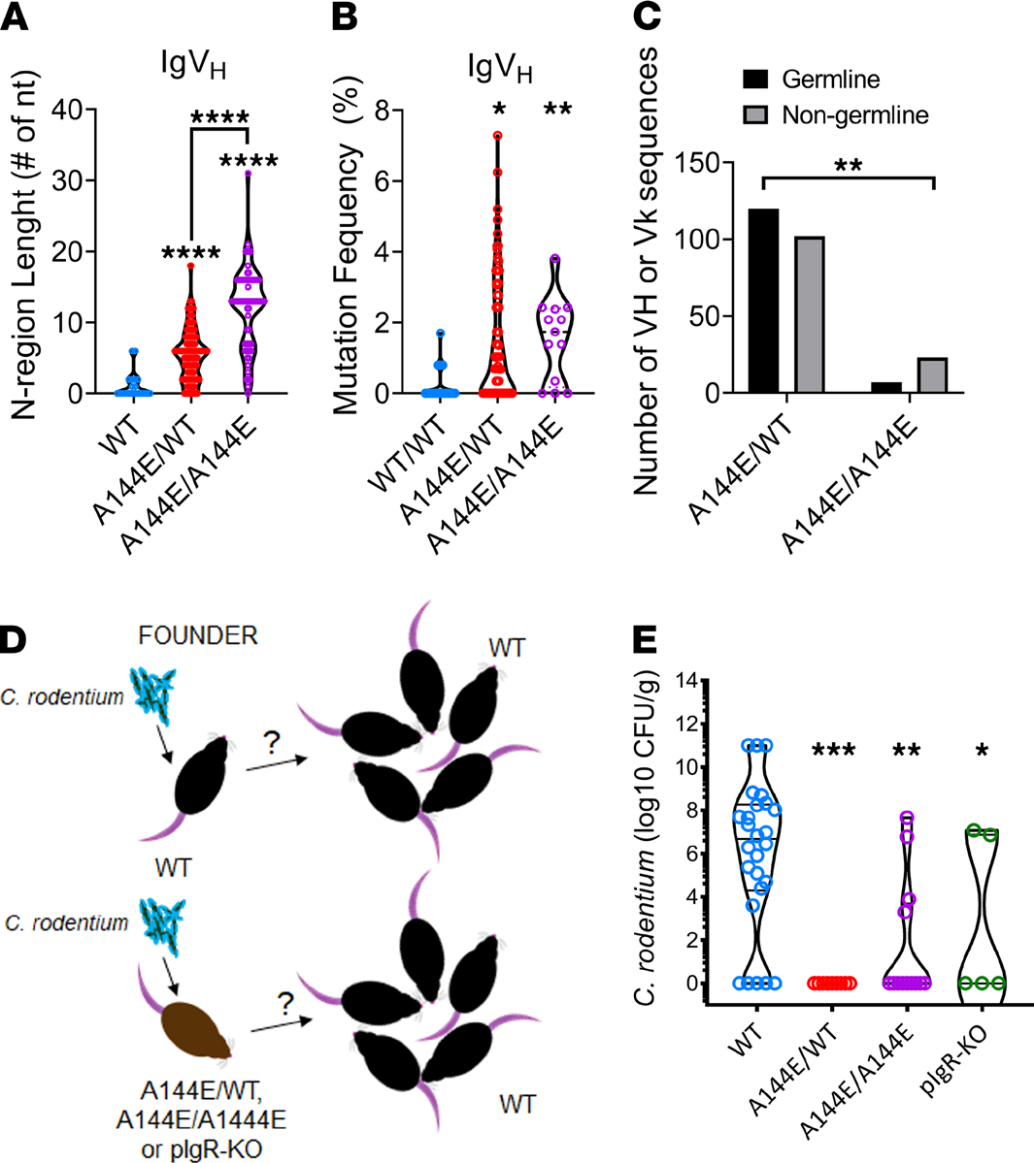
IgA sequences from sorted *C. rodentium*–bound IgA^+^ B cells of C57BL/6 and *Tnfrsf13b* mutant mice have distinct properties and determine *C. rodentium* spreading. Single IgA^+^ GFP-*C*. *rodentium*–bound B cells were sorted from the Peyer’s patches of infected C57BL/6, A144E/WT, or A144E/A144E mice 14 days following primary infection (**A–C**) (See also Supplemental Figure 5 and Supplemental Tables 1–6). IgA H+L sequences were obtained from cDNA by PCR followed by Sanger sequencing or by next generation sequencing of barcoded single cell barcoded cDNA libraries. (**A**) Graphs compare the lengths of the N regions (number of nts) of CDR3 regions (in amino acids) of HC IgA sequences obtained from C57BL/6 A144E/WT or A144E/A144E mice. The Kruskal-Wallis test followed by Dunn’s multiple comparisons test yielded P < 0.0001 in comparison of the N region lengths in mutant mice with those in WT mice. (**B**) Graphs depict the frequencies (%) of mutated nts in VH or Vκ exons relative to their closest germline in HC sequences obtained from C57BL/6, A144E/WT, or A144E/A144E mice. The Kruskal-Wallis test followed by Dunn’s multiple comparisons test yielded *P* < 0.0001 in comparison of the N region lengths in mutant mice with those in WT mice, and yielded *P* = 0.0094 or *P* = 0.0195 in comparisons of the frequencies of VH gene mutations in A144E/A144E mutants or A144E/WT mice, to those in WT mice, respectively. (**C**) Contingency analysis of frequency of germline *C*. *rodentium* binding IgA, VH, and Vκ sequences obtained from A144E/WT or A144E/A144E mice. The χ^2^ test yielded a *P* = 0.016, indicating that germline sequences are rarer in A144E/A144E mice compared with A144E/WT mice. (**D**) Schematic of experimental protocol. Founder mice are either C57BL/6, *Tnfrsf13b* mutant mice, or PIgR-KO mice. Two founder mice were cohoused with 3 C57BL/6 mice/cage. Only founders were inoculated with low virulence 10^8^ *C*. *rodentium*. CFUs were counted on not primarily infected mice 7 days after infection. (**E**) Graph depicts CFU/g feces in mice not primarily infected according to the strain of the inoculated founders (depicted in the *x* axis). Comparisons were by Kruskal-Wallis tests followed by Dunn’s multiple comparison tests. The Kruskal-Wallis test yielded *P* < 0.0001; Dunn’s multiple comparison tests to WT yielded *P = 0.0111, **P = 0.0060, ***P = 0.0004, ****P < 0.0001.

To determine what IgA properties were attributable to the *Tnfrsf13b* genotype as opposed to those determined by the environment, we sequenced IgA (H+L) from single cells using 10× GEM technology obtained from WT, A144E/WT, or A144E/A144E littermates, 14 days after infection. Figure 4, A and B, and Supplemental Tables 5 and 6 largely confirm that the A144E allele dose is associated with larger N regions and decreased frequencies of germline sequences. These results show that *Tnfrsf13b* mutations decreased the frequency of germline Ig sequences (more frequent among natural antibodies) produced in response to *C*. *rodentium* infection; however, *Tnfrsf13b* mutant mice can mount ample T cell–dependent B cell responses to *C*. *rodentium*.

### *Tnfrsf13b* A144E or PIgR-KO alleles control *C. rodentium* spreading.

*C*. *rodentium* might exploit germline-encoded natural IgA to secure a niche and prolong residence in the gut. However, this adaptation would be eclipsed if germline-encoded IgA hindered transmission of virulent organisms. To evaluate this possibility, we examined the transmissibility of *C*. *rodentium* from WT and from various mutant strains of mice to uninfected susceptible WT mice (Figure 4, D and E). WT or *Tnfrsf13b* mutant mice were infected with *C*. *rodentium* (founder mice), and cohoused mice were tested for infection 7 days later. WT mice transmitted disease to 21 of 25 cohoused WT mice (84%). In contrast, monoallelic A144E mice transmitted *C*. *rodentium* to no other cohoused mice, whether WT or mutant. Biallelic A144E transmitted *C*. *rodentium* to only 4 of 13 cohoused WT mice (30.8%) and PIgR-KO transmitted infection to 2 of 5 (40%) cohoused WT mice. Thus, natural IgA not only induces virulence, it also enables transmission of *C*. *rodentium* within a colony. Our results suggest the extraordinary frequency *Tnfrsf13b* mutations might in part have been preserved to counter this vulnerability.

## Discussion

Here, we report what may constitute an example of balancing selection and coevolution. Balancing selection ideally requires: (a) variant alleles in which each variant is maintained at a particular equilibrium frequency, (b) a distinct benefit afforded by some fraction of the variants and/or heterozygous advantage, and (c) a biological cost. In one famous example of balancing selection, the sickle cell β-globin gene is maintained in populations because the heterozygous state affords resistance to malaria at the cost of homozygote-associated sickle cell disease (27). We show in this report that *Tnfrsf13b* mutant alleles that impair receptor function enhance resistance to *C*. *rodentium* and limit bacterial dissemination because of decreased natural IgA, which *C*. *rodentium* has coopted to express virulence.

Like the sickle cell β-globin gene the advantage given by *TNFRSF13B* mutants comes with a biological cost. Indeed, *TNFRSF13B* mutants are associated with common variable immune deficiency (CVID) (5) and autoimmunity (28). In contrast to the sickle cell β-globin gene, *TNFRSF13B* polymorphisms govern many facets of immunity. We propose that *TNFRSF13B* polymorphisms define a continuum of immune responses by controlling aspects of both innate and adaptive immunity. We (1, 2) and others (29) showed that *Tnfrsf13b* governs differentiation of plasma cells, controls the synthesis of “natural IgA antibodies” (showed herein), and determines affinity maturation of antibodies by controlling functions of B and Th follicular cells in the germinal center (4). Whereas the sickle cell β-globin gene impacts only the carrier, *Tnfrsf13b* polymorphisms may impact on the health of the community by limiting transmission of bacteria regulated by *LEE*-like loci. Therefore, our findings suggest that the high frequency of dominant-negative *TNFRSF13B* variants may be maintained by balancing selection.

Commensal organisms compete favorably against and hence suppress *C*. *rodentium* late in the course of infection (30). However, gut commensals are also required for *C*. *rodentium* colonization of the mucosa (31). We found the composition of the microbiota to significantly differ between *Tnfrsf13b* mutant and WT mice (Supplemental Figures 6 and 7) but that in itself did not explain the relative resistance to infection by *Tnfrsf13b* mutant mice given that: (a) normalization of the microbiota by cohousing (for 4 weeks) did not render *Tnfrsf13b* mutant mice susceptible or WT mice resistant to *C*. *rodentium* infection; (b) transfer of feces SN from WT mice, which do not contain bacteria, to resistant *Tnfrsf13b* mutant mice induces susceptibility; and (c) progeny of WT or *Tnfrsf13b* mutant mice manifest the susceptibility associated with their genotype (not the susceptibility associated with their mothers). Thus, A144E heterozygous mice, obtained by crossing WT mice with homozygous *Tnfrsf13b* A144E mice, frequently resist infection or produce 20-fold less CFUs than WT littermates, and *Tnfrsf13b* A144E/A144E and *Tnfrsf13b*-KO mice, produced and backcrossed to C57BL/6 independently, are resistant. These facts support the resistant phenotype is a direct consequence of *Tnfrsf13b* genotype.

The prevailing concept is that IgA is protective (21, 32, 33). IgA is thought to protect against pathogens by exerting direct effects such as neutralizing virulence (decreasing motility by binding to adhesins, pili, and flagella) (34–36) by promoting entrapment in the mucus layer (37), immune exclusion (38), clearance by agglutination and enchained growth (9, 39), or by direct modulation of gene expression (40, 41). In one example, natural IgA protects against *Salmonella typhimurium* and against necrotizing enterocolitis in newborn infants (42, 43). IgA may also protect against pathogens by indirect actions such as enhancing colonization by protective (antiinflammatory) commensal species (44). For example, *Bacteroides fragilis* capsule induces specific IgA, which in turn, increases its adherence to intestinal epithelial cells (33), and commensals, such as *Bacteroides thetaiotaomicron,* use IgA to support mutualism (41). However, our data indicate that *Tnfrsf13b* mutants induce resistance to *C*. *rodentium* because of a relative depletion of IgA “natural” antibodies in the gut, which induce *C*. *rodentium* virulence. Accordingly, WT sterile feces SN (and IgA alone) induce virulence, in accord to their IgA concentration. Mouse natural IgA (evoked against C-carbohydrate from *Streptococcus pneumoniae,* purchased from Southern Biotech, catalog 0106-01) bound *C*. *rodentium* and induced virulence in a concentration-dependent manner. Furthermore, pIgR-deficient mice that hardly have any IgA in feces, owing to an independent mechanism, are resistant to *C*. *rodentium*. We have also determined that TACI deficiency confers protection against infection with uropathogenic *E*. *coli* (our unpublished observations). This is the first report to our knowledge in which the pathogens’ adaptation to “natural” IgA to increase virulence and transmissibility has been described. Because *Tnfrsf13b* mutants maintain the ability to make “adaptive” IgA it is possible that protective functions of IgA are maintained.

For all the advantages IgA provides, IgA deficiency is the most common immune deficiency, with frequencies varying between 1:143 in the Arabian Peninsula to 1 in 500 White individuals (45, 46). This number may be underestimated since IgA deficiency is often asymptomatic. One of our studies (5, 47) showed that all of the patients with *TNFRSF13B* mutations and common variable immunodeficiency examined also had IgA deficiency, and that 1 IgA-deficient patient also had a *TNFRSF13B* mutation. In a recent study, Pulvirenti et al. (48) showed that 13% of IgA-deficient patients carried at least 1 mutated *TNFRSF13B* allele. Most IgA-deficient individuals are asymptomatic, and only a small percentage develop recurrent sinopulmonary infections and/or autoimmune manifestations (45). Given the limited morbidity of IgA deficiency, one might wonder whether benefits conferred by constraint on virulence of *LEE*-dependent enterobacteria outweigh the detrimental impact of decreased IgA in the gut. Data showing high frequency of H and L mutations in *C*. *rodentium–*specific B cells in mutant mice suggest enhanced (compensatory) adaptive antibody responses. In accord, IgA-deficient patients were found to have enhanced adaptive antibody responses to pneumococcal vaccination (49). It is perhaps this type of response that explains the mild phenotype of many individuals with IgA deficiency.

In addition to controlling mucosal IgA, *TNFRSF13B* mutant alleles also enhance inflammatory responses owing to decreased IgM and IgG natural antibodies. In another study, we show (our unpublished observations) are associated with antibody-mediated rejection in human subject recipients of kidney transplants owing to enhanced inflammatory responses and accelerated complement deposition. Consistent with this possibility we found that *Tnfrsf13b* mutant mice produced less “natural” antibodies and exhibited enhanced complement deposition in endogenous kidneys. Although maladaptive in response to transplantation, our findings suggest that the enhanced inflammatory responses in *Tnfrsf13b* mutant mice and in human subjects expressing *TNFRSF13B* variants might be adaptive in response to certain microbes.

We show here that a common *Tnfrsf13b* variant, even when expressed from a single allele together with the WT gene, induces resistance to an enterohemorrhagic microbe by blocking expression of virulence genes, which in turn limit transmission and dissemination of disease. *C*. *rodentium* is a commonly used model for enteropathogenic infections in humans, dependent on the *LEE* locus. Given the pronounced impact of monoallelic mutations, our findings identify a receptor that might be temporarily targeted to disrupt transmission of organisms in epidemics of organisms regulated by *LEE*-type loci.

## Methods

### Experimental models and subject details

#### Mice.

C57BL/6 WT mice were purchased from The Jackson Laboratory (C57BL/6J, IMSR catalog JAX:000664). PIgR-KO mice (50), *Tnfrsf13b*-KO mice (6), and mice harboring bi- (A144E/A144E) or monoallelic A144E variants (WT/A144E) (16), *Tnfrsf13b*, homologous to the human A181E, were previously described. A144E/A144E mice were maintained by breeding males and females of the same genotype. WT/A144E mice were obtained by breeding A144E/A144E males with C57BL/6 females or A144E/A144E females with C57BL/6 males. All of the KO mice and *Tnfrsf13b* mutant mice were bred into the C57BL/6 background. In some experiments, A144E/WT and WT littermates were obtained from crosses between WT mothers and A144E/WT males or from crosses between A144E/WT mothers and WT males. Animals of both sexes between 8–20 weeks of age were maintained under specific pathogen–free conditions.

#### C. rodentium.

The kanamycin-resistant (Km-resistant) WT *C*. *rodentium* strain DBS120 (pCRP1:Tn5) was a gift from David Schauer, Massachusetts Institute of Technology, Cambridge, Massachusetts, USA (51). The *C*. *rodentium* expressing GFP (52), used to identify *C*. *rodentium*–bound B cells, was a gift from Bruce Vallance, University of British Columbia, Vancouver, Canada. The *C*. *rodentium* expressing a plasmid containing a *ler/lux* transcriptional fusion was previously described (12). Bacteria were grown overnight in Luria-Bertani (LB) broth supplemented with Km (50 μg/mL) with agitation at 225 rpm at 37°C. To produce high and low virulence inoculum, 1 mL bacteria suspension was added into either 9 mL autoclaved LB with Km (low virulence) or to 9 mL DMEM (high virulence). Those cultures were incubated in agitation at 37°C for 6 more hours, bringing both culture concentrations to 10^8^ CFU/mL.

#### Infections.

Mice were infected by oral gavage with 0.2 mL PBS containing approximately 10^8^ CFU. At the designated time points, the feces were collected, weighed, suspended in 1 mL PBS, and serially diluted. Optimally diluted feces were plated on MacConkey plates and cultured at 37°C overnight. The number of colonies was counted, and the number of bacteria per 1 g feces was calculated. Infection was considered cleared when no colonies were detected in the undiluted feces suspension. In some experiments, mice were given naive C57BL/6 feces supernatants by gavage (200 μL) twice, 24 hours before infection and 2 days after infection. Feces SN sterility was ensured by centrifugation followed by culture in LB agar to verify absence of colonies or by filtering through a 0.2 μm filter.

#### Measurement of ler expression.

*ler* expression was determined by measuring luminescence emitted by *ler/lux*-expressing bacteria in PBS suspensions, or in gut tissue ex vivo. Luminescence was detected with bioluminescence imaging (BLI) using an IVIS200 (Xenogen Corporation). When measuring luminescence of bacteria attached to the intestinal wall, the entire gastrointestinal tract was removed, bisected, washed with PBS, and placed into the light-tight chamber of the charge-coupled device camera system of the IVIS 200 immediately. For PBS suspensions of feces and mouse IgA and IgG (Southern Biotech, catalog 0106-01, RRID:AB_2714214 and catalog 0107-01, RRID:AB_2732898, respectively), 5 *×* 10^5^ expressing *C*. *rodentium* were incubated for 1 hour at room temperature with feces supernatant serial dilutions or different concentrations of mouse IgA.

Luminescence emitted from *lux*-expressing bacteria in the tissue was quantified using the Living Image Software v.4.7.2 (IVIS Imaging Systems, Xenogen Corporation, RRID:SCR_014247). Relative luminescence units were obtained by dividing the total light measured in photons/second per unit of area in 10 minutes and dividing that number by the background luminescence, followed by standardization for the weight of feces when supernatants were used. Real-time quantitative PCR (qPCR) for *ler* was performed using a SYBR green PCR master mix to confirm that luminescence reflected *ler* expression according to Kamada et al. (12). Briefly, the expressions of *ler* and *Km*-resistant protein were detected by qPCR using 5′-AATATACCTGATGGTGCTCTTG-3′ and 5′-TTCTTCCATTCAATAATGCTTCTT-3′, and 5′-CTGAATGAACTGCAGGACGA-3′ and 5′-ATACTTTCTCGGCAGGAGCA-3′, respectively, and the expression of the virulence factor *ler* was normalized to the expression of *Km*-resistant protein. The relative expression of *ler* was determined as fold change when compared with the expression of bacteria cultured in LB medium.

#### Detection of total C. rodentium–reactive Igs.

To detect mouse Ig, Nunc MaxiSorp ELISA plates were coated for 1 hour at room temperature with goat anti-mouse Ig (H+L) (4 μg/mL; Southern Biotech, catalog 1010-01, RRID: AB_2794121). *C*. *rodentium*–specific Ig was quantified by coating Nunc MaxiSorp ELISA plates with 10^8^ bacteria/well heat-inactivated at 60°C for 1 hour. After blocking, the plates were incubated with serial dilutions of mouse sera or feces supernatant for 1 hour at room temperature. Bound IgG, IgM, or IgA was detected by adding HRP-conjugated goat anti-mouse IgG (4 μg/mL; Southern Biotech, catalog 1030-05, RRID: AB_2619742), goat anti-mouse IgM (4 μg/mL; Southern Biotech, catalog 1020-05, RRID: AB_2794201), or goat anti-mouse IgA (4 μg/mL; Southern Biotech, catalog 1040-05, RRID: AB_2714213). The reaction was visualized by subsequent addition of 2,2′-Azino-bis (3-ethylbenzothiazoline-6-sulfonic acid) substrate (Southern Biotech, catalog 0202-01).

#### Flow cytometry, sorting, and antibodies.

In Peyer’s patches, the lymphocytes were isolated from naive or infected mice as reported by Tsuji et al. and Cascalho et al. (1, 53). Cell viability was assessed via BD Horizon Fixable Viability Stain 780 (FVS780, 1.11 μg/mL; BD Biosciences, catalog 565388), and cells were stained with APC rat anti-mouse CD19 (1D3; 10 μg/mL; BD Biosciences, catalog 550992, RRID: AB_39848), PE rat anti-mouse IgA (mA-6E1, 4 μg/mL; Thermo Fisher Scientific, catalog 12-4204-82, RRID: AB_465917), and *C*. *rodentium* expressing GFP. Staining was performed in 10^6^ cells, data were acquired with a BD FACS Canto II (BD Biosciences), and 100,000 events were analyzed with FlowJo v.10.6.1 (RRID:SCR_008520). Single cell sorting was done on 96-well plates using a FACS Aria II (BD Biosciences) in the Biomedical Research Facilities Core at the University of Michigan.

To quantify IgA binding to *C*. *rodentium* in noninfected mice, feces supernatants were diluted at 10 mg/mL in PBS, and binding of feces IgA to *C*. *rodentium* was detected by flow cytometry. Briefly, 10^8^ *C*. *rodentium* expressing GFP were incubated with various dilutions of feces supernatant for 30 minutes at 4°C. Bacteria were then washed, and bound IgA was detected with a PE-conjugated rat anti-mouse IgA (mA-6E1, 4 μg/mL; Thermo Fisher Scientific, catalog 12-4204-82, RRID: AB_465917). Data were acquired with a BD FACS Canto II (BD Biosciences), 100,000 events were recorded in the *C*. *rodentium*-GFP gate, and were analyzed with FlowJo v.10.6.1 (RRID:SCR_008520).

#### Ig gene sequencing.

*IgA^+^ GFP-C. rodentium^+^* B cells obtained from the Peyer’s patches of mice infected with *C*. *rodentium* 14 days earlier or 5 days after reinfection were single-cell sorted into of U-bottom 96-well plates according to methods adapted from Tiller et al. (54).Briefly, cells were sorted into PCR plates containing 4 μL/well of ice-cold 0.5× PBS supplemented with 10 mM DTT (Invitrogen, catalog Y00147), 8 U RNasin Ribonuclease Inhibitor (Promega, catalog N2115), and 3 U Recombinant RNase Inhibitor (Takara, catalog 2313A), sealed and immediately frozen on dry ice. Total RNA from single-sorted B cells was reverse transcribed in the original sorting plate with 150 ng Random Hexamer Primer (Thermo Scientific, catalog S0142), 1 mM dNTP Mix (Invitrogen, catalog 18080044), 7 mM DTT (Thermo Scientific, catalog P2325), 0.5% v/v IGEPAL CA-630 (Sigma-Aldrich, catalog I3021-50ML), 4 U RNasin Ribonuclease Inhibitor (Promega, catalog N2115), 6 U Recombinant RNase Inhibitor (Takara, catalog 2313A), 50 U Superscript III reverse transcriptase (Invitrogen, catalog 18080-044), and nuclease-free water in a final volume of 14 μL/well. Reverse transcription was performed at 42°C for 5 minutes, at 25°C for 10 minutes, 50°C for 60 minutes, and 94°C for 5 minutes. cDNA was stored at −20°C. Mouse *Igh*, *Igk,* and *Igl* V gene transcripts were amplified by 2 rounds of semi-nested (*Igh*) or nested (*Igk* and *Igl*) PCR in 96-well plates containing 200 nM each primer or total primer mix (Supplemental Table 7), 300 μM dNTP Mix (Invitrogen, catalog 18080044), and 1.2 U HotStart Taq DNA polymerase (Qiagen, catalog 203205). The first round of reactions was performed with 3.5 μL cDNA at 94°C for 15 minutes followed by 50 cycles of 94°C for 30 seconds, 56°C (*Igh*) or 50°C (*Igk*) or 58°C (*Igl*) for 30 seconds, 72°C for 55 seconds, and final incubation at 72°C for 10 minutes. After identification of the light chain genes the semi-nest or nested second-round PCR was performed with 3.5 μL of first-round PCR product as template and combinations of V, J, and C primers (Supplemental Table 7) at 94°C for 15 minutes followed by 50 cycles of 94°C for 30 seconds, 60°C (*Igh*) or 45°C (*Igk*) or 58°C (*Igl*) for 30 seconds, 72°C for 45 seconds, and final incubation at 72°C for 10 minutes. PCR products were treated with ExoSAP-IT PCR Product Cleanup Reagent (Applied Biosystems, catalog 78201.1.ML) and sequencing was performed by the Sanger method at the University of Michigan Sequencing Core. Analysis of the sequences was done by using the IMGT portal (55–57) (RRID:SCR_011812), alignments were by Multiple sequence alignment by Log-expectation, MUSCLE software (58, 59) (RRID:SCR_011812). Comparisons of the amino acid composition of *C*. *rodentium*–specific IgA HC was done using the Kullback-Leibler logotype using the Seq2Logo 2.0 software (60) (http://www.cbs.dtu.dk/biotools/Seq2Logo/index.php). Primer sequences are described on Supplemental Table 8. Alternatively, IgA^+^ GFP-*C*. *rodentium^+^* B cells were sorted as explained previously in PBS 0.004% BSA, and up to 10,000 cells were analyzed by Chromium Next Gel Bead-in-Emulsions (GEM) Single Cell V(D)J Technology. Briefly, cells were identified via generation of GEMs by combining barcoded Single Cell V(D)J 5’ Gel Beads v1.1, a master mix with cells (Chromium Next GEM Single Cell 5′ Library and Gel Bead Kit v1.1; 10× Genomics catalog 1000165), and partitioning Oil on Chromium Next GEM Chip G (10× Genomics catalog 1000127), and reverse transcription and cDNA amplification were performed as recommended by the manufacturer. Next, the targeted enrichment from cDNA was conducted with the Chromium Single Cell V(D)J Enrichment Kit, Mouse B Cell (10× Genomics, catalog 1000072). The cDNA quality control analysis was carried out in an Agilent 2100 Bioanalyzer (Agilent Technologies) using the Agilent High Sensitivity DNA Kit (Agilent Technologies, catalog 5067-4626). The V(D)J enriched library was then constructed via Chromium Single Cell 5’ Library Construction Kit (10× Genomics, catalog 1000020), and libraries were sequenced in a NovaSeq 6000 Sequencing System (Illumina). V(D)J sequences were collapsed using Cell Ranger: V(D)J Pipelines (10× Genomics, RRID:SCR_017344), and the V usage and clonotype profiles were generated and visualized by Loupe VDJ Browser. We were able to recover between 200 to more than 8,000 cell barcodes per sample.

#### 16S RNA sequencing.

DNA was extracted from feces pellets using the Qiagen MagAttract PowerMicrobiome DNA/RNA EP kit (QIAGEN, catalog 27500-4-EP). The V4 region of the 16S rRNA-encoding gene was amplified from extracted DNA using the barcoded dual-index primers developed by Kozich et al. (61) (Supplemental Table 8). Samples were amplified using AccuPrime Taq DNA Polymerase, high fidelity (Invitrogen, catalog 12346086) at 95°C for 2 minutes followed by 30 cycles of 95°C for 20 seconds, 55°C for 15 seconds, and 72°C for 5 minutes, and final incubation at 72°C for 10 minutes, purified using a magnetic bead capture kit (Agencourt AMPure; Beckman Coulter, catalog 000130) and quantified using a fluorometric kit (Quant-iT PicoGreen dsDNA Assay Kit; Invitrogen, catalog P7589). Purified amplicons were pooled in equimolar concentrations with a SequalPrep Normalization Plate Kit (Applied Biosystems, catalog A1051001) and sequenced on Illumina MiSeq System (RRID:SCR_016379). Bioinformatic analysis was done using the Mothur v.1.42.3 software package (62) (RRID:SCR_011947) available at the University of Michigan Microbial Systems Laboratory.

#### Data availability.

The published study includes all data sets analyzed during this study.

#### Statistics.

All comparisons were done with GraphPad Prism v.8.0.0 software (RRID:SCR_002798). When an assumption of normal distributions could not be made, values in more than 2 groups were compared using the Kruskal-Wallis test followed by a Dunn’s multiple comparison test. Comparisons of 2 groups were done by the Mann-Whitney test or the Wilcoxon test for paired analysis. When an assumption of Gaussian distribution could be made, averages were compared by unpaired 2-tailed *t* test or when comparing more than 2 groups, using 1-way ANOVA followed by multiple comparisons tests. Correlations were determined by the Spearman’s rank test. Differences in bacterial community structure were analyzed using analysis of molecular variance in Mothur v.1.42.3 (62) (RRID:SCR_011947). Data are shown as mean ± SEM. A *P* value of equal or less than 0.05 was considered significant. Further information about which the statistical tests used in each experiment and experimental number can be found in the figure legends.

#### Study approval.

All the experiments were performed in accordance with the approved animal protocol and the regulations of University of Michigan Committee on the Use and Care of Animals.

## Author contributions

MC and JLP conceptualized the project. Methodology, MC and JLP performed methodology. MGMB, DH, ARL, JK, CK, CMB, and CW supervised the experiments. MC wrote the original draft of the manuscript. MC, MGMB, CMB, CW, RG, RB, GN, NK, and JLP participated in the review and editing of the manuscript. MC and JLP supervised funding acquisition for the project. MC, CMB, and JLP managed the resources. MC and JLP supervised the project.

## Supplementary Material

Supplemental data

## Version 1 06/10/2021

In-Press Preview

## Version 2 07/22/2021

Electronic publication
