# Supplementary material for: TNFRSF13B polymorphisms counter microbial adaptation to enteric IgA
Source: JCI Insight. 2021 Jul 22;6(14):e148208. doi: 10.1172/jci.insight.148208 (PMC8410086; doi:10.1172/jci.insight.148208)
Supplement: Supplemental data [file jciinsight-6-148208-s169.pdf]

Supplementary Materials for

Natural IgA and *TNFRSF13B* polymorphism: a double edged sword fueling balancing selection

Authors: Jeffrey L. Platt[1*], Mayara Garcia de Mattos Barbosa[2], Daniel Huynh[2], Adam R. Lefferts[2], Juhi Katta[2], Cyra Kharas[2], Peter L Freddolino[3], Christine M. Bassis[4], Christiane Wobus[5], Raif Geha[6], Richard Bram[7], Gabriel Nunez[8], Nobuhiko Kamada[4] and Marilia Cascalho[1*]

Correspondence to: marilia@med.umich.edu

This PDF file includes:

Supplementary Text
Figs. S1 to S7
Tables S1 to S8

Supplementary text:

Data and Materials Availability

Further information and requests for resources and reagents should be directed to and will be fulfilled by the Lead Contact, Marilia Cascalho (marilia@med.umich.edu). This study did not generate new unique reagents. Data not included in the main text such as microbiota S16 sequences and IgA sequences will be made available on the Sequence Read Archive-NCBI data base.

Natural antibodies in *Tnfrsf13b* mutant mice prior to infection

Consistent with prior reports (*3*), *tnfrsf13b*-mutant mice had decreased "natural" IgM but as much or more IgG in the blood than their wild type controls (Figure S1A).

***Tnfrsf13b* mutant mice mount recall responses**

Productive infection and/or *C. rodentium*-specific IgG titer predict the outcome of a secondary infection. Thus, mice that had a productive primary infection with peak CFU/g of feces $>10^5$ and/or developed anti-*C. rodentium* IgG titers above 10^{-4} during primary challenge resist secondary infection independently of the genotype (Figures S2F-G). Thus, *Tnfrsf13b* genotype governs baseline resistance and immunity to *C. rodentium.* The mechanisms of resistance and immunity are distinct. While baseline resistance to *C. rodentium* does not depend on acquired immunity; enhanced clearance upon re-infection is clearly dependent on acquired immunity.

Microbiota composition in *Tnfrsf13b* mutant mice

To determine how *Tnfrsf13b*-mutant alleles shaped the microbiota, we performed 16S RNA sequencing on DNA extracted from stool samples derived from wild type or *Tnfrsf13b*-mutant mice before and following *C. rodentium* infection. Results depicted in figures S6 and S7 A and B show that *Tnfrsf13b*-mutant mice express a more diverse microbiota than wild type mice prior to infection and the difference in diversity disappears with infection principally due to changes in the microbiota in wild type mice. Differences in microbiota composition do not explain resistance to *C. rodentium* since resistance persists after co-housing for 4 weeks and is exhibited by A144E/WT and not by WT/WT littermates.

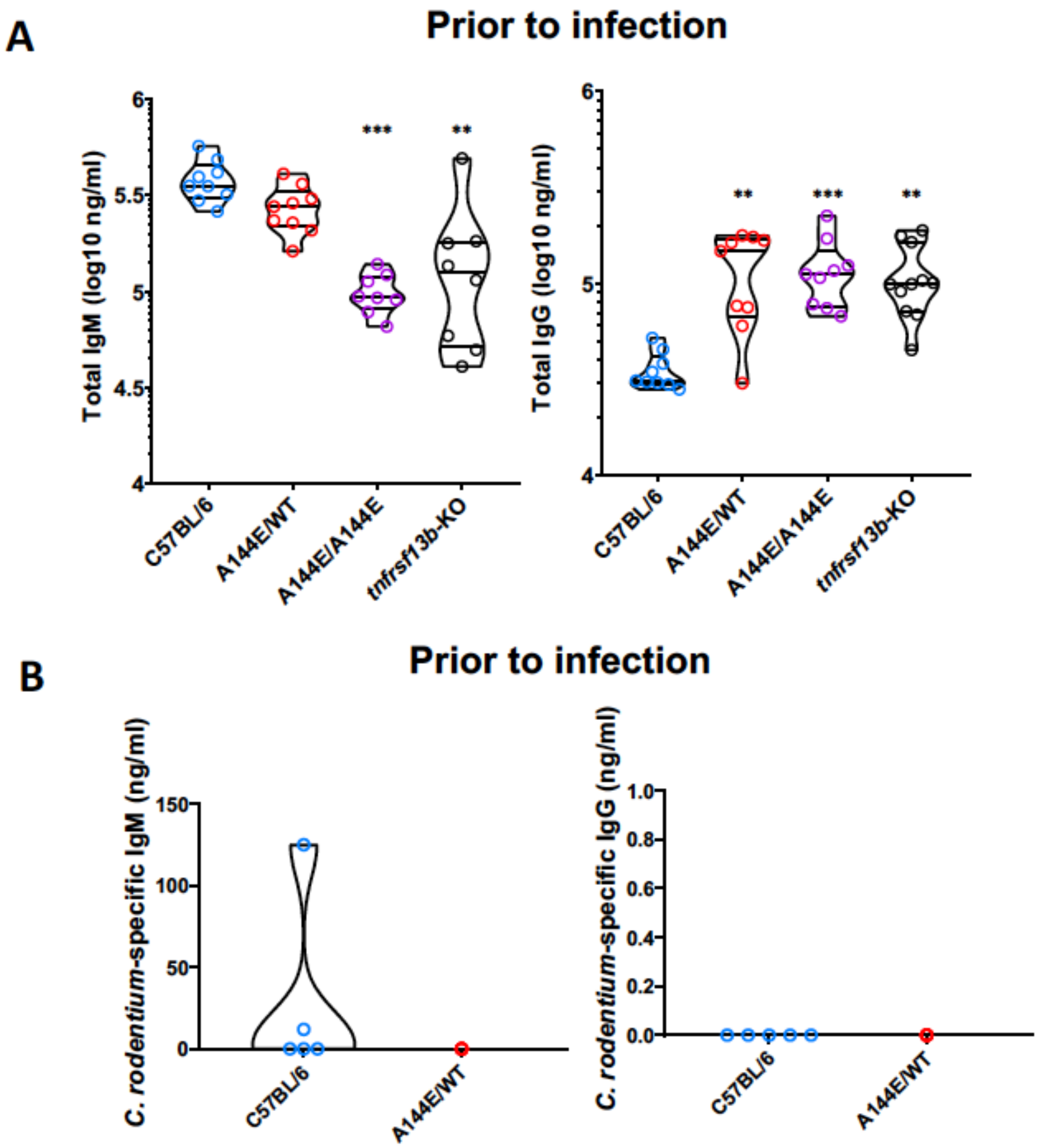

Fig. S1. Analysis of total and *C. rodentium* or intimin-specific IgM and IgG before infection. (A and B) Graphs represent the total IgM (left) and total IgG (right) concentrations (log10 ng/ml) in the blood before infection. Analysis was by One-way ANOVA followed by Dunnett's multiple comparisons tests comparing concentrations of IgG and IgM in *Tnfrsf13b*-mutant mice with those in C57BL/6 mice. ANOVA yielded a $p<0.0001$ (IgM) or $p=0.8198$ (IgG). Dunnett's multiple comparisons tests comparing mutants to WT yielded $p=0.1121$-IgM and $p=0.7509$-IgG, A144/WT; $p<0.0001$-IgM, $p=0.5465$-IgG, A144E/A144E; $p<0.0001$-IgM, $p=0.3028$-IgG, *tnfrsf13b*-KO. (B) Graphs represent the concentration of *C. rodentium*-specific IgM (left) or IgG (right) in blood (Y-axis) in WT or A144/WT mice prior to infection.

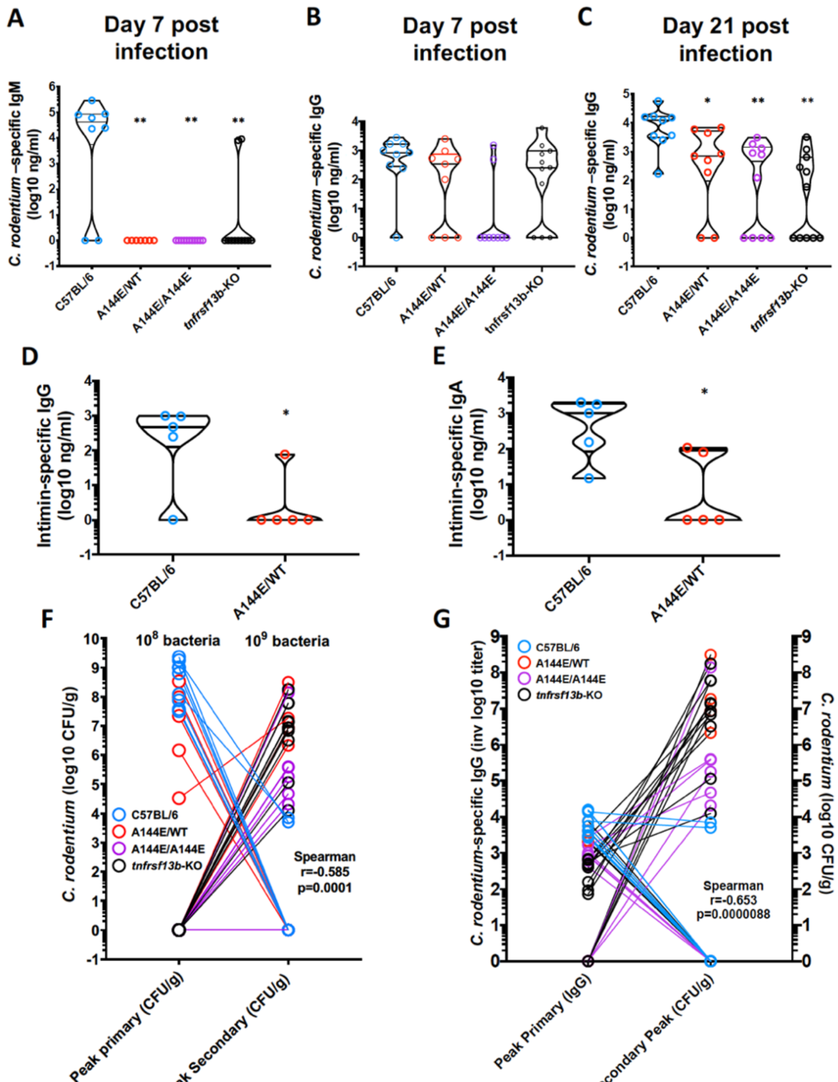

Figure S2

Fig. S2. Primary infection and *C. rodentium*-specific antibodies protect mice from secondary infection. C57BL/6 mice and mice with *Tnfrsf13b* A144E mutations or *Tnfrsf13b*-KO were administered 10^8 *C. rodentium* by oral gavage and levels of antibodies against *C. rodentium* were assayed by ELISA. Anti-*C. rodentium* IgM and IgG were measured 7 days after infection; anti *C. rodentium* IgG, anti-intimin IgG or anti-intimin IgA 21 days later, a time when T cell-dependent IgG responses to *C. rodentium* achieve maximum affinity. None of the mice had detectable IgM, IgG or IgA specific for *C. rodentium* or specific for intimin in the serum prior to infection (See also Figure S1), hence the results shown reflect responses to infection. (A) Anti-*C. rodentium* IgM in serum; (B-C) *C. rodentium*-specific IgG in the blood of mice 7 days (B) or 21 days (C) after infection. Only A144E/A144E mice had significantly decreased *C. rodentium*-specific IgG. Dunnett's multiple comparisons tests yielded $p<0.01$. (D) Anti-intimin IgG in serum. (E) Anti-intimin IgA in serum. (F) Relationship between maximum CFU/g of feces in the course of primary infection with CFU/g of feces 5 days after secondary infection. (G) Relationship between anti-*C. rodentium* IgG titer in serum and CFU/g of feces of *C. rodentium* in stool 5 days after re-infection. The results indicate that having a productive primary infection or *C. rodentium*-specific IgG titer of 10^{-3} or below protects against a secondary challenge. Correlations were analyzed by the Spearman test and indicate that CFU/g of feces during primary infection or anti-*C. rodentium* IgG titer and CFUs after re-infection are inversely correlated.

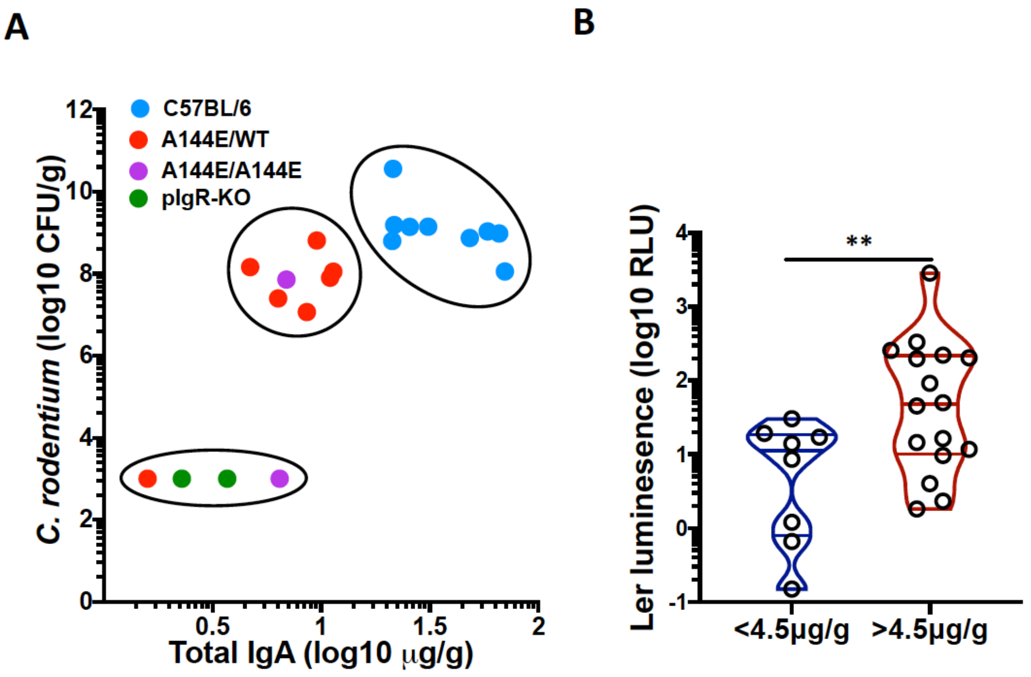

Figure S3

Fig. S3. ***Ler* expression and CFU increase with IgA concentration in the gut and IgA concentration in the gut is determined by the genotype.** (A) IgA concentration determines CFU at 7 days post-infection. The groups reflecting the IgA concentrations in relation to CFU outcomes have been noted in the figure with oval lines. (B) *Ler* expression in bacteria attached to gut walls of mice that have less than 4.5 µg of IgA per gram of feces is significantly decreased in comparison with *ler* expression in bacteria attached to gut walls of mice that have more than 4.5 µg of IgA per gram of feces. Analysis with the Mann-Whitney test yielded P=0.0029, one-tailed.

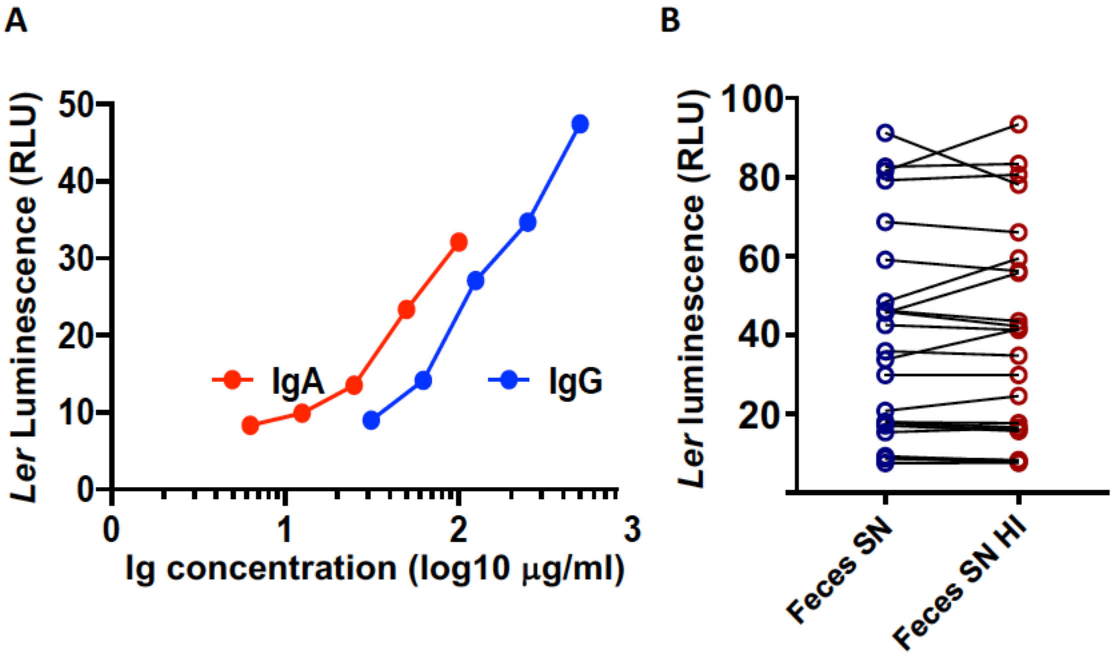

Figure S4

Fig. S4: IgA and IgG of irrelevant specificity induce *ler* when added to *C. rodentium* and induction of *ler* is independent of heat-labile complement. (A) Murine IgG or IgA purchased from Southern Biotech (cat#01101-01 and #01107-01, respectively) or (B) WT feces supernatant before and after heat inactivation at 56°C for 30 minutes were added to *C. rodentium* in culture. Luminescence (*ler-lux* luminescence, Y-axis) was detected with bioluminescence imaging (BLI) using an IVIS200 (Xenogen Corporation, Alameda, CA). Figure shows that heat inactivation does not inhibit virulence induction.

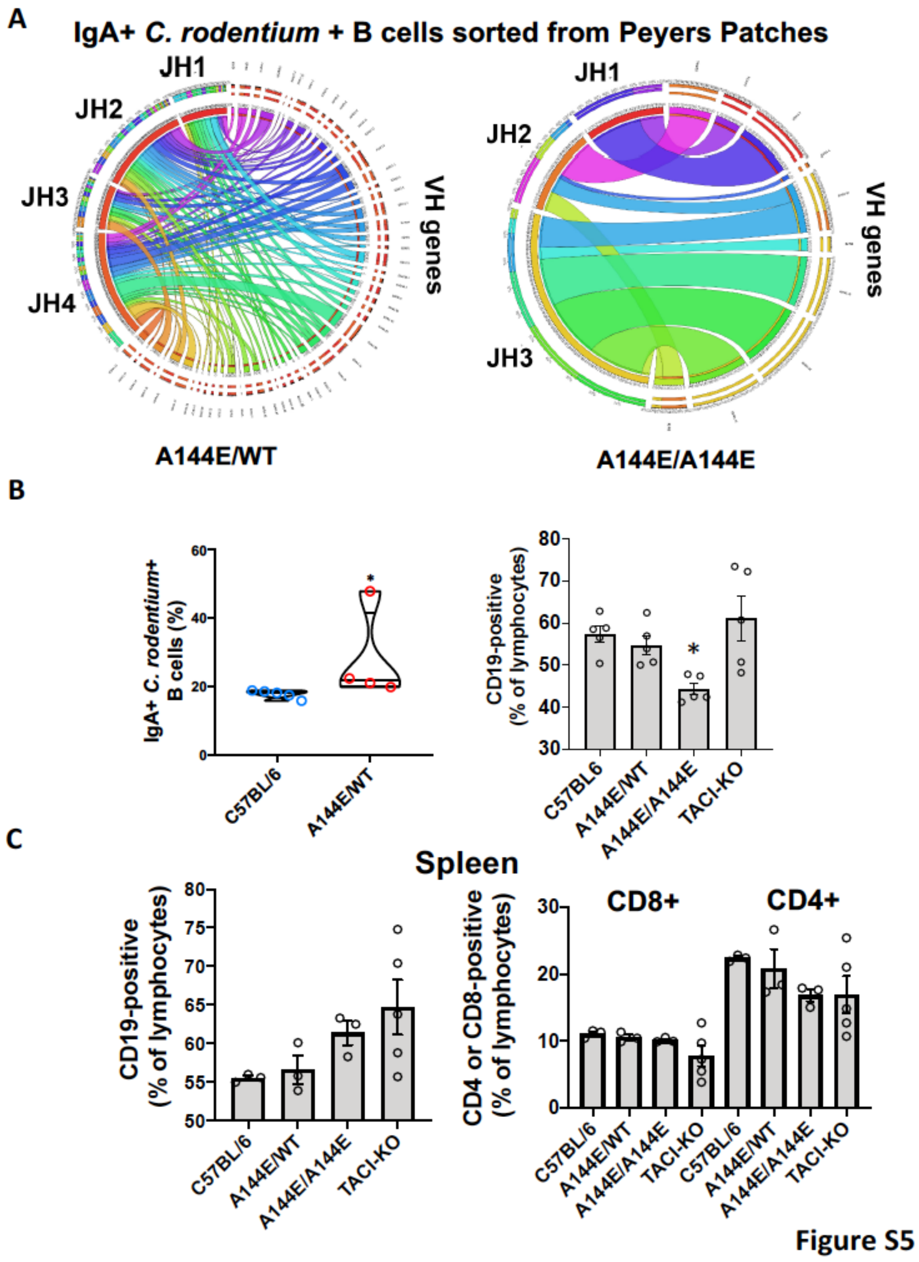

Fig S5. Flow cytometry analysis of lymphocytes 14 days after infection and IgH repertoire diversity circos plots of Peyer's patches IgA-positive, *C. rodentium*-positive B cells. (A) Circos plots obtained from VH gene sequences isolated from mice 14 days following infection with 10^8 *C. rodentium.* (B) Flow cytometry analysis of IgA-positive *C. rodentium*-positive B cells isolated from Peyer patches. (C) Frequency of CD19-positive CD4-positive and CD8-positive lymphocytes in spleens of infected mice.

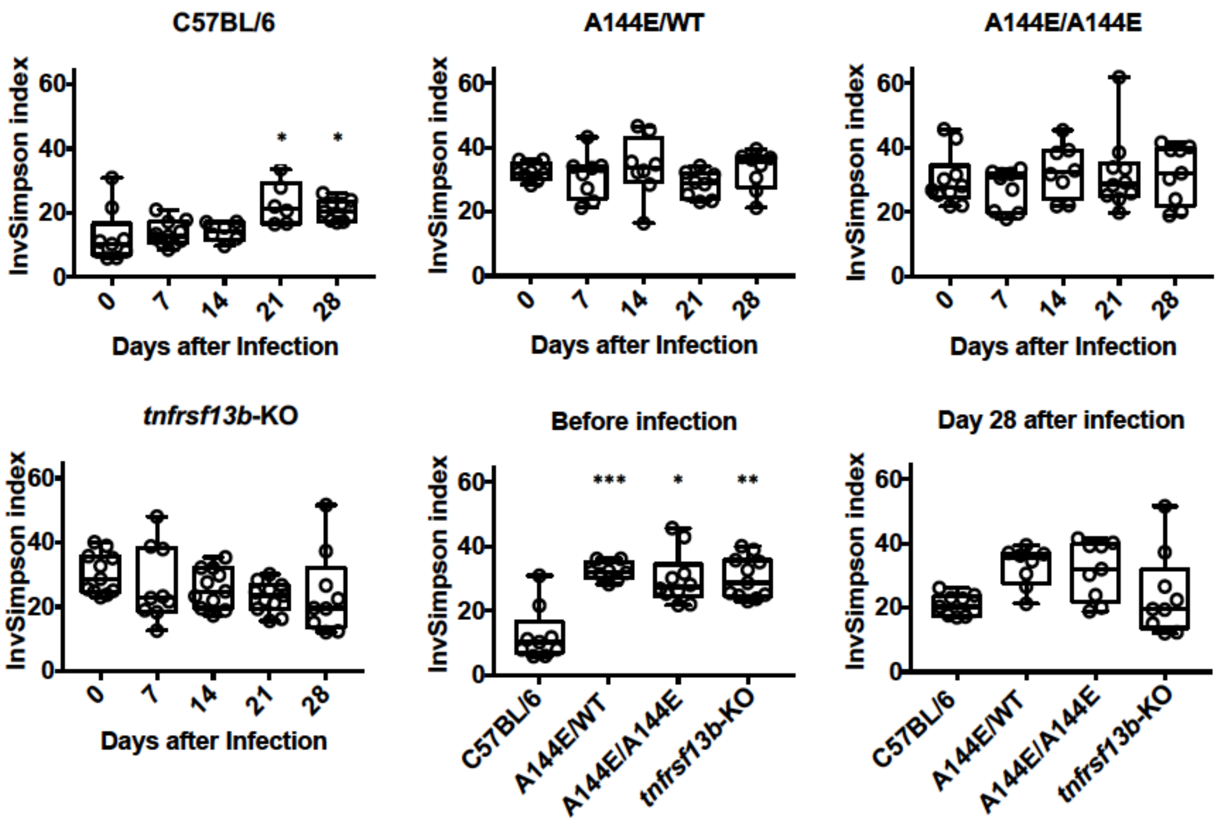

Figure S6

Fig.S6. Microbiota diversity. Species diversity measured using the InvSimpson Index. Measurements were determined before and weekly after infection with *C. rodentium*. Figure shows that *Tnfrsf13b*-mutant mice have greater microbiota diversity compared to C57BL/6 mice before infection but not after. Diversity only changes in C57BL/6 mice following infection. Analysis comparing diversity in *Tnfrsf13b*-mutant mice and C57BL/6 mice was by the Kruskal-Wallis test. p<0.05 *, p<0.01**, p<0.001*

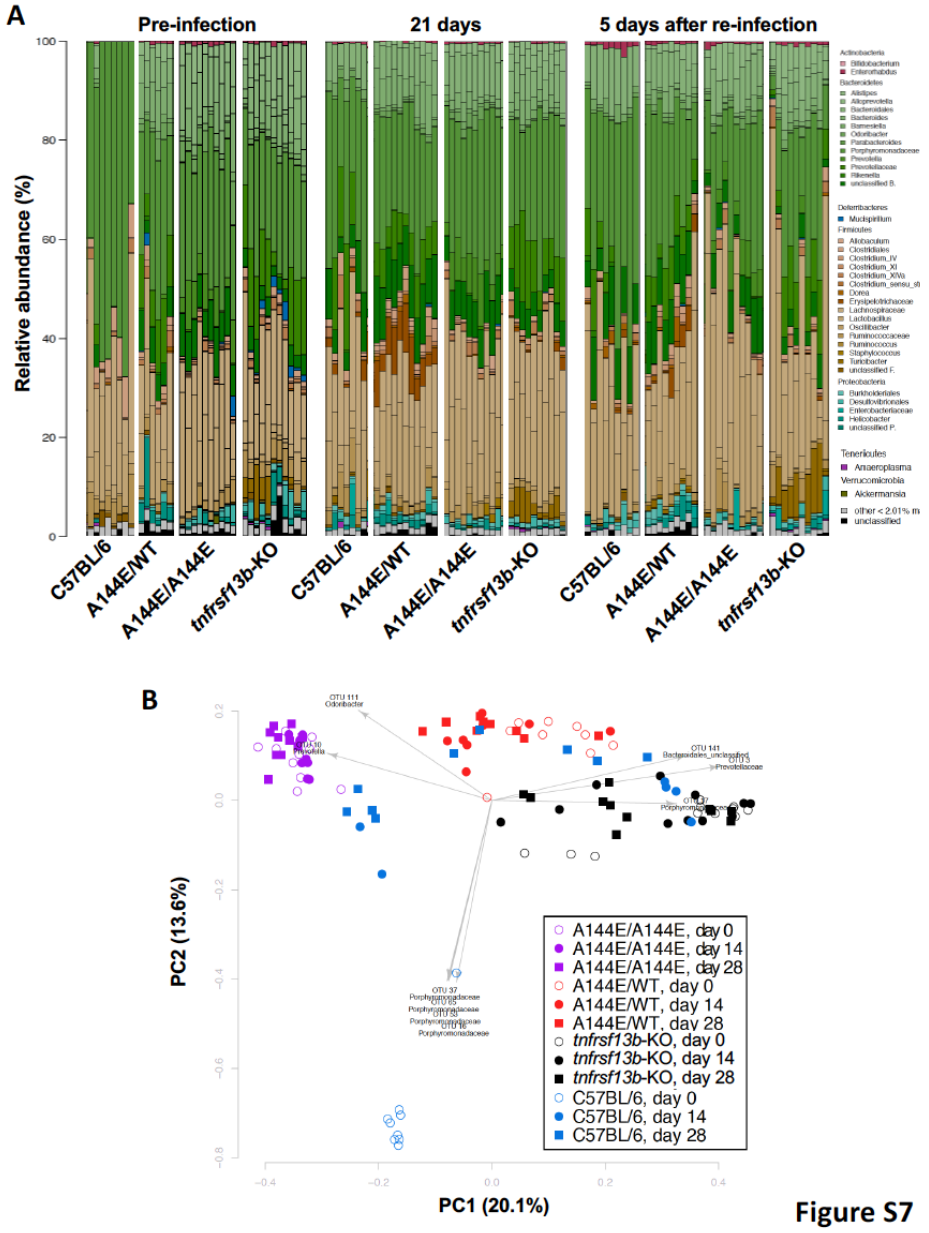

Fig.S7. Microbiota composition. (A) Schematic of the species composition in mice before and after *C. rodentium* infection. (B) Principal Component Analysis of microbiota composition at different times after infection. Analysis shows that microbiota in *Tnfrsf13b*-mutant mice remains clustered throughout infection while microbiota in C57BL/6 mice change dramatically.

Tables:

Table S1: Immunoglobulin clones from C57BL/6 IgA-positive *C. rodentium*-bound B cells sorted from Peyer's patches

ID	Clone	Count	Frequency (%)	CDR3 nucleotide sequence	CDR3 amino acid sequence	Productive	Chain Type	V gene	D gene	J gene
**1**	**B4IgH_C03**	3	33.3	ATTAGGGGGGACTACT TTGACTAC	IRGDYFDY	Yes	VH	IGHV5-17*01	IGHD3-3*01	IGHJ2*01
**2**	**D3IgH_C09**	2	22.2	GCAAGAACTGGGACG AACTACTTTGACTAC	ARTGTNYF DY	Yes	VH	IGHV1-9*01	IGHD4-*01, IGHD4-1*02	IGHJ2*01
**3**	**B4IgH_F08**	1	11.1	GCAATCGGGGACGAG GGCTAC	AIGDEGY	Yes	VH	IGHV1-74*01, IGHV1-74*04	IGHD3-*01, IGHD4-1*01, IGHD4-1*02	IGHJ2*01
**4**	**D3IgH_C12**	1	11.1	GCAGGAGGCCTGGGG GGCTAC	AGGLGGY	Yes	VH	IGHV1-85*01	IGHD4-1*01, IGHD4-1*02	IGHJ2*01
**5**	**D3IgH_C03**	1	11.1	GCGAGCGGCCATCTA CTATGTTACGTGCCTG GTTTGCTTAC	ASGHLLCY VPGLLT	Yes	VH	IGHV3-3*01, IGHV3-3*02	IGHD2-1*01, IGHD2-13*01, IGHD2-2*01	IGHJ3*01
**6**	**B4IgH_G05**	1	11.1	GTACGAGAGACTCCCT ACTACTTTGACTCC	VRETPYYF DS	Yes	VH	IGHV1-80*01	IGHD6-2*01, IGHD6-2*02	IGHJ2*01

Table S2: Identity and query names of the immunoglobulin clones from C57BL/6 IgA-positive *C. rodentium*-bound B cells sorted from Peyer's patches.

ID	Count	Frequency (%)	Minimum Identity (%)	Maximum Identity (%)	Average Identity (%)	All Query Name(s)
**1**	3	33.3	95.9	99.2	97.9	B4IgH_C03, B4IgH_D03, B4IgH_D04
**2**	2	22.2	98.6	100	99.3	D3IgH_C09, D3IgH_D09
**3**	1	11.1	98.3	98.3	98.3	B4IgH_F08
**4**	1	11.1	92.8	92.8	92.8	D3IgH_C12
**5**	1	11.1	92.9	92.9	92.9	D3IgH_C03
**6**	1	11.1	91.4	91.4	91.4	B4IgH_G05

Table S3: Immunoglobulin clones from A144E/A144E IgA-positive *C. rodentium*-bound B cells sorted from Peyer's patches

ID	Clone	Count	Frequency (%)	CDR3 nucleotide sequence	CDR3 amino acid sequence	Productive	Chain Type	V gene	D gene	J gene
**1**	**C3IgH_D09**	1	10	GCAAGAAGGGGACATTACTACGATAGTAGCTACTATGCTTTGGACTAC	ARRGHYYDSSYYALDY	Yes	VH	IGHV1-9*01	IGHD1-1*01	IGHJ4*01
**2**	**C3IgH_F05**	1	10	GCAAGACCCGCCTACTATAGTAAAGAGTTTGCGTAC	ARPAYYSKEFAY	Yes	VH	IGHV1-9*01	IGHD2-5*01, IGHD2-6*01	IGHJ3*01
**3**	**C3IgH_D01**	1	10	GCAAGACGGCTCAGTACTACGGTAGTCCCTTTTGCTTAC	ARRLSTTVVPFAY	Yes	VH	IGHV1-56*01	IGHD1-1*01	IGHJ3*01
**4**	**A5IgH_F02**	1	10	GCAAGAGAAGAGGCTGATGGTTCGGGGATTGCTCAC	AREEADGSGIAH	Yes	VH	IGHV3-6*01	IGHD2-3*01	IGHJ3*01
**5**	**A5IgH_B02**	1	10	GCAAGGCGGGACAGCTCAGGCTCCTGGTTTGCTTAC	ARRDSSGSWFAY	Yes	VH	IGHV1-47*01	IGHD3-2*02	IGHJ3*01
**6**	**A5IgH_F04**	1	10	GCAAGGGGTGGTTACTACTGGTACTTCGATGTC	ARGGYYWYFDV	Yes	VH	IGHV5-17*01	IGHD1-1*02, IGHD2-2*01, IGHD2-3*01	IGHJ1*03
**7**	**C3IgH_G07**	1	10	GCCAGAAGGTTCTGGATCAGGAGCTACTGGTACTTCGATGTC	ARRFWIRSYWYFDV	Yes	VH	IGHV2-9-1*01	IGHD3-2*02	IGHJ1*03
**8**	**A5IgH_F06**	1	10	GCCAGAGAGGGGGCGGGAGGGAGGTTTGCTTAC	AREGAGGRFAY	Yes	VH	IGHV2-5*01, IGHV2-5-1*01	N/A	IGHJ3*01
**9**	**A5IgH_H02**	1	10	GTGAGAGATAGGGGCTATTACTACGGTAGTAGCTACCCCTACTATGCTATGGACTAC	VRDRGYYYGSSYPYYAMDY	Yes	VH	IGHV10-3*01	IGHD1-1*01	IGHJ4*01
**10**	**A5IgH_H11**	1	10	GTTATTTTTCCCGGCTATTCTTTGGACTAC	VIFPGYSLDY	Yes	VH	IGHV14-1*01, IGHV14-4*02	IGHD1-1*01, IGHD1-1*02	IGHJ4*01

Table S4: Identity and query names of the immunoglobulin clones from A144E/A144E IgA-positive *C. rodentium*-bound B cells sorted from Peyer's patches.

ID	Count	Frequency (%)	Minimum Identity (%)	Maximum Identity (%)	Average Identity (%)	All Query Name(s)
**1**	1	10	94.7	94.7	94.7	C3IgH_D09
**2**	1	10	93.3	93.3	93.3	C3IgH_F05
**3**	1	10	99.6	99.6	99.6	C3IgH_D01
**4**	1	10	91.6	91.6	91.6	A5IgH_F02
**5**	1	10	99.2	99.2	99.2	A5IgH_B02
**6**	1	10	99.6	99.6	99.6	A5IgH_F04
**7**	1	10	96.2	96.2	96.2	C3IgH_G07
**8**	1	10	95.3	95.3	95.3	A5IgH_F06
**9**	1	10	100	100	100	A5IgH_H02
**10**	1	10	93.9	93.9	93.9	A5IgH_H11

Table S5: Identity and query names of the Immunoglobulin heavy chain clones from A144E/WT or A144E/A144E IgA-positive *C. rodentium*-bound B cells sorted from Peyer's patches.

	Genotype	CDR3-IMGT length (AA)	CDR3-IMGT sequence (AA)	V gene and allele	D gene and allele	J gene and allele	Anchors 104,118	V %	Seq. length	Function	#
1	A144E/A144E	17 AA	VRGALYYYGSSHSWFAY	Musmus IGHV1-80*01 F	Musmus IGHD1-1*01 F	Musmus IGHJ3*01 F	C104,F118	97.57	603	productive	1
2	A144E/A144E	16 AA	ARREDYYGSSLSWFAY	Musmus IGHV1-9*01 F	Musmus IGHD1-1*01 F	Musmus IGHJ3*01 F	C104,F118	98.26	601	productive	1
3	A144E/A144E	15 AA	ARSEVYYGSSYWFAY	Musmus IGHV1-81*01 F	Musmus IGHD1-1*01 F	Musmus IGHJ3*01 F	C104,F118	100	600	productive	1
4	A144E/A144E	15 AA	TRGDYYGTSYVEFAY	Musmus IGHV1-76*01 F	Musmus IGHD1-1*01 F	Musmus IGHJ3*01 F	C104,F118	97.57	605	productive	1
5	A144E/A144E	14 AA	ARSIYYGSSGYFDY	Musmus IGHV1-81*01 F	Musmus IGHD1-1*01 F	Musmus IGHJ2*01 F	C104,F118	100	589	productive	1
6	A144E/A144E	14 AA	ARSYYAGIYWFFDV	Musmus IGHV3-3*01 F	Musmus IGHD1-1*01 F	Musmus IGHJ1*03 F	C104,F118	98.61	598	productive	1
7	A144E/A144E	13 AA	ASIRYGNYWYFDV	Musmus IGHV3-6*01 F	Musmus IGHD2-1*01 F	Musmus IGHJ1*03 F	C104,F118	97.92	562	productive	1
8	A144E/A144E	8 AA	AHSGLFAY	Musmus IGHV1-26*01 F	Musmus IGHD3-1*01 F	Musmus IGHJ3*01 F	C104,F118	98.61	581	productive	2
9	A144E/A144E	8 AA	LHSGLFAY	Musmus IGHV1-26*01 F	Musmus IGHD1-1*02 F	Musmus IGHJ3*01 F	C104,F118	97.92	577	productive	1
10	A144E/A144E	7 AA	ARLGGYP	Musmus IGHV1-54*01 F	Musmus IGHD2-2*01 F	Musmus IGHJ2*01 F	C104,F118	100	666	productive	1
11	A144E/A144E	7 AA	TTWWFAY	Musmus IGHV6-6*01 F	Musmus IGHD3-1*01 F	Musmus IGHJ3*01 F	C104,F118	97.62	689	productive	1

	Genotype	CDR3-IMGT length (AA)	CDR3-IMGT sequence (AA)	V gene and allele	D gene and allele	J gene and allele	Anchors 104,118	V %	Seq. length	Function	#
		**ARSGAFRGY FDV**									
1	A144E/A144E	12 AA	ARSGAFRGYFDV	Musmus IGHV1-54*01 F, or Musmus IGHV1-54*02 F	Musmus IGHD2-3*01 F	Musmus IGHJ1*03 F	C104,F118	99.65	565	productive	1
	A144E/WT	12 AA	ARSGAFRGYFDV	Musmus IGHV1-54*01 F, or Musmus IGHV1-54*02 F	Musmus IGHD2-3*01 F	Musmus IGHJ1*03 F	C104,F118	99.65	567	productive	1
		**ATNWDVFAY**									
2	A144E/A144E	9 AA	ATNWDVFAY	Musmus IGHV1-53*01 F	Musmus IGHD4-1*01 F	Musmus IGHJ3*01 F	C104,F118	96.18	557	productive	1
	A144E/WT	9 AA	ATNWDVFAY	Musmus IGHV1-53*01 F	Musmus IGHD4-1*01 F	Musmus IGHJ3*01 F	C104,F118	96.18	547	productive	1

	Genotype	CDR3-IMGT length (AA)	CDR3-IMGT sequence (AA)	V gene and allele	D gene and allele	J gene and allele	Anchors 104,118	V %	Seq. length	Function	#
1	A144E/WT	18 AA	AREGIFYYGSKEGYYFDY	Musmus IGHV1-55*01 F	Musmus IGHD1-1*01 F	Musmus IGHJ2*01 F	C104,F118	100	607	productive	1
2	A144E/WT	17 AA	ARTYYYGSSYEGYYFDY	Musmus IGHV1-63*01 F	Musmus IGHD1-1*01 F	Musmus IGHJ2*01 F	C104,F118	100	546	productive	1
3	A144E/WT	17 AA	ARWGYHGSSYVRNYFDY	Musmus IGHV1-80*01 F	Musmus IGHD1-1*01 F	Musmus IGHJ2*01 F	C104,F118	98.26	615	productive	1
4	A144E/WT	17 AA	VRERGGYNYDEADYFDY	Musmus IGHV5-4*01 F	Musmus IGHD2-12*01 F	Musmus IGHJ2*01 F, or Musmus IGHJ2*02 F	C104,F118	96.53	623	productive	1
5	A144E/WT	16 AA	ARWHGYDEEDYYTMDY	Musmus IGHV1-80*01 F	Musmus IGHD2-2*01 F	Musmus IGHJ4*01 F	C104,F118	98.96	604	productive	1
6	A144E/WT	16 AA	ARWHGYDEGDYYTMDF	Musmus IGHV1-80*01 F	Musmus IGHD2-2*01 F	Musmus IGHJ4*01 F	C104,F118	98.96	608	productive	1
7	A144E/WT	16 AA	ARWHGYDEGDYYTMDY	Musmus IGHV1-80*01 F	Musmus IGHD2-2*01 F	Musmus IGHJ4*01 F	C104,F118	98.96	603	productive	2
8	A144E/WT	16 AA	ARWLGYDEGDYYAMDY	Musmus IGHV1-80*01 F	Musmus IGHD2-2*01 F	Musmus IGHJ4*01 F	C104,F118	99.31	603	productive	1
9	A144E/WT	16 AA	ARWLGYDEGDYYSMDY	Musmus IGHV1-80*01 F	Musmus IGHD2-2*01 F	Musmus IGHJ4*01 F	C104,F118	98.96	676	productive	1
10	A144E/WT	15 AA	ARGEDIYYGRSLGDY	Musmus IGHV1-54*01 F	Musmus IGHD1-1*01 F	Musmus IGHJ2*01 F, or Musmus IGHJ2*02 F	C104,F118	97.57	599	productive	1
11	A144E/WT	15 AA	ARRNYYGSSYWYFDV	Musmus IGHV1-69*01 F, or Musmus IGHV1-69*02 F	Musmus IGHD1-1*01 F	Musmus IGHJ1*03 F	C104,F118	97.22	601	productive	1
12	A144E/WT	15 AA	ARRNYYSSSYWYFDV	Musmus IGHV1-69*01 F, or Musmus IGHV1-69*02 F	Musmus IGHD1-1*01 F	Musmus IGHJ1*03 F	C104,F118	96.53	598	productive	2
13	A144E/WT	15 AA	ARSGGYGGGYWFFDV	Musmus IGHV1-75*01 F	Musmus IGHD1-1*02 F	Musmus IGHJ1*03 F	C104,F118	92.71	603	productive	1
14	A144E/WT	15 AA	ARWRDDYDPYWYFDV	Musmus IGHV1-9*01 F	Musmus IGHD2-4*01 F	Musmus IGHJ1*03 F	C104,F118	100	697	productive	1
15	A144E/WT	15 AA	TRDIYYGSDYYAMDY	Musmus IGHV5-9-1*02 F	Musmus IGHD1-1*01 F	Musmus IGHJ4*01 F	C104,F118	100	695	productive	1
16	A144E/WT	14 AA	ARDYGSTYWGYFDV	Musmus IGHV1-52*01 F	Musmus IGHD1-1*01 F	Musmus IGHJ1*03 F	C104,F118	100	661	productive	1
17	A144E/WT	14 AA	ARGDYGSSYWYFDV	Musmus IGHV1-64*01 F	Musmus IGHD1-1*01 F	Musmus IGHJ1*03 F	C104,F118	99.65	596	productive	4
18	A144E/WT	14 AA	ARGGTTIAPSAMDY	Musmus IGHV1-37*01 F	Musmus IGHD1-1*01 F	Musmus IGHJ4*01 F	C104,F118	95.49	596	productive	1
19	A144E/WT	14 AA	ARGGTTIVPSAMDY	Musmus IGHV1-37*01 F	Musmus IGHD1-1*01 F	Musmus IGHJ4*01 F	C104,F118	96.53	631	productive	5
20	A144E/WT	14 AA	ARGGTTVVPSAMDY	Musmus IGHV1-37*01 F	Musmus IGHD1-1*01 F	Musmus IGHJ4*01 F	C104,F118	97.22	597	productive	1
21	A144E/WT	14 AA	ARGLYYGSPYAMDY	Musmus IGHV1-39*01 F	Musmus IGHD1-1*01 F	Musmus IGHJ4*01 F	C104,F118	100	687	productive	1
22	A144E/WT	14 AA	ARIKYYGSYWYFDV	Musmus IGHV8-8*01 F	Musmus IGHD1-1*01 F	Musmus IGHJ1*03 F	C104,F118	100	559	productive	1
23	A144E/WT	14 AA	ARPYYGSSYYAMDY	Musmus IGHV9-3*01 F	Musmus IGHD1-1*01 F	Musmus IGHJ4*01 F	C104,F118	98.26	600	productive	1
24	A144E/WT	14 AA	ARRITTVVAGSMDY	Musmus IGHV1-18*01 F	Musmus IGHD1-1*01 F	Musmus IGHJ4*01 F	C104,F118	100	703	productive	1

**25**	A144E/WT	14 AA	ARSDWNDYVWFFDV	Musmus IGHV1-64*01 F	Musmus IGHD2-4*01 F	Musmus IGHJ1*01 F, or Musmus IGHJ1*03 F	C104,F118	96.88	580	productive	1
**26**	A144E/WT	14 AA	ARSDWYDYVWFFDV	Musmus IGHV1-64*01 F	Musmus IGHD2-4*01 F	Musmus IGHJ1*01 F, or Musmus IGHJ1*03 F	C104,F118	95.83	582	productive	1
**27**	A144E/WT	14 AA	ARSDWYEYVWFFDV	Musmus IGHV1-64*01 F	Musmus IGHD2-4*01 F	Musmus IGHJ1*01 F, or Musmus IGHJ1*03 F	C104,F118	95.83	596	productive	1
**28**	A144E/WT	14 AA	ARSEYYGSKGYFDV	Musmus IGHV1-81*01 F	Musmus IGHD1-1*01 F	Musmus IGHJ1*03 F	C104,F118	100	688	productive	1
**29**	A144E/WT	14 AA	ARSWLLLYLYSMDY	Musmus IGHV1-53*01 F	Musmus IGHD2-12*01 F	Musmus IGHJ4*01 F	C104,F118	98.61	590	productive	2
**30**	A144E/WT	14 AA	ARSYYHGSFYAMDY	Musmus IGHV3-3*01 F	Musmus IGHD1-1*01 F	Musmus IGHJ4*01 F	C104,F118	99.31	598	productive	2
**31**	A144E/WT	14 AA	ARSYYHGSFYSMDY	Musmus IGHV3-3*01 F	Musmus IGHD1-1*01 F	Musmus IGHJ4*01 F	C104,F118	98.96	607	productive	1
**32**	A144E/WT	14 AA	ARSYYYGSFYTLDY	Musmus IGHV3-3*01 F	Musmus IGHD1-1*01 F	Musmus IGHJ4*01 F	C104,F118	98.96	600	productive	1
**33**	A144E/WT	14 AA	GRGGTTVVPSAMDY	Musmus IGHV1-37*01 F	Musmus IGHD1-1*01 F	Musmus IGHJ4*01 F	C104,F118	95.83	597	productive	1
**34**	A144E/WT	14 AA	VKAPPYYSNSWFAY	Musmus IGHV7-4*01 F	Musmus IGHD2-5*01 F	Musmus IGHJ3*01 F	C104,F118	98.64	622	productive	1
**35**	A144E/WT	13 AA	AGRITTVVGAMDY	Musmus IGHV1-12*01 F	Musmus IGHD1-1*01 F	Musmus IGHJ4*01 F	C104,F118	98.26	602	productive	1
**36**	A144E/WT	13 AA	ARHEERYYYAMDY	Musmus IGHV1-62-2*01 F		Musmus IGHJ4*01 F	C104,F118	100	684	productive	1
**37**	A144E/WT	13 AA	ARIASSVFYYFDY	Musmus IGHV8-8*01 F	Musmus IGHD3-2*02 F	Musmus IGHJ2*01 F	C104,F118	99.31	559	productive	1
**38**	A144E/WT	13 AA	ARPGGYDGWYFDV	Musmus IGHV1-78*01 F	Musmus IGHD2-2*01 F	Musmus IGHJ1*03 F	C104,F118	100	564	productive	1
**39**	A144E/WT	13 AA	ARQELTGYYAMDY	Musmus IGHV5-15*01 F	Musmus IGHD4-1*01 F	Musmus IGHJ4*01 F	C104,F118	99.31	614	productive	1
**40**	A144E/WT	13 AA	ARRDYGSGGYFDV	Musmus IGHV1-66*01 F	Musmus IGHD1-1*01 F	Musmus IGHJ1*03 F	C104,F118	100	677	productive	1
**41**	A144E/WT	13 AA	ARRDYYGSPYFDY	Musmus IGHV1-26*01 F	Musmus IGHD1-1*01 F	Musmus IGHJ2*01 F	C104,F118	100	684	productive	1
**42**	A144E/WT	13 AA	ARSGYNNYDWFAY	Musmus IGHV1-53*01 F	Musmus IGHD2-5*01 F	Musmus IGHJ3*01 F	C104,F118	97.57	594	productive	2
**43**	A144E/WT	13 AA	ARSSGSSLYYFDY	Musmus IGHV1-19*01 F	Musmus IGHD1-1*01 F	Musmus IGHJ2*01 F	C104,F118	98.61	590	productive	1
**44**	A144E/WT	13 AA	VRIASSVFYYFDY	Musmus IGHV8-8*01 F	Musmus IGHD3-2*02 F	Musmus IGHJ2*01 F	C104,F118	98.28	649	productive	1
**45**	A144E/WT	12 AA	AGDKLGYWYFDV	Musmus IGHV12-3*01 F	Musmus IGHD4-1*01 F	Musmus IGHJ1*03 F	C104,F118	99.31	533	unproductive	1
**46**	A144E/WT	12 AA	AGDRDGYWYFDV	Musmus IGHV12-3*01 F	Musmus IGHD2-3*01 F	Musmus IGHJ1*03 F	C104,F118	100	375	productive	3
**47**	A144E/WT	12 AA	AGDSS*PDAMDY	Musmus IGHV12-3*01 F	Musmus IGHD1-1*01 F	Musmus IGHJ4*01 F	C104,F118	100	680	unproductive	1
**48**	A144E/WT	12 AA	AKHEERDYAMDY	Musmus IGHV2-9*01 F	Musmus IGHD2-14*01 F	Musmus IGHJ4*01 F	C104,F118	100	721	productive	1
**49**	A144E/WT	12 AA	ARGAYYSNYGGY	Musmus IGHV1-85*01 F	Musmus IGHD2-5*01 F	Musmus IGHJ2*01 F	C104,F118	100	578	productive	1
**50**	A144E/WT	12 AA	ARGGPYYGAMDY	Musmus IGHV1-78*01 F	Musmus IGHD1-1*01 F	Musmus IGHJ4*01 F	C104,F118	100	651	productive	1

**51**	A144E/WT	12 AA	ARHDYSNFYFDY	Musmus IGHV1-62-2*01 F	Musmus IGHD2-5*01 F	Musmus IGHJ2*01 F	C104,F118	100	591	productive	1
**52**	A144E/WT	12 AA	ARIAGDPPWFAY	Musmus IGHV8-8*01 F	Musmus IGHD1-3*01 F	Musmus IGHJ3*01 F	C104,F118	100	645	productive	2
**53**	A144E/WT	12 AA	ARLVGFRGYFDV	Musmus IGHV9-3*01 F	Musmus IGHD1-1*02 F	Musmus IGHJ1*03 F	C104,F118	93.75	564	productive	1
**54**	A144E/WT	12 AA	ARMRDRGYYFDY	Musmus IGHV2-9-1*01 F	Musmus IGHD2-3*01 F	Musmus IGHJ2*01 F	C104,F118	100	683	productive	1
**55**	A144E/WT	12 AA	ARRGDYDGAMDY	Musmus IGHV1-12*01 F	Musmus IGHD2-4*01 F	Musmus IGHJ4*01 F	C104,F118	98.96	592	productive	1
**56**	A144E/WT	12 AA	ARYEGLLQGFDY	Musmus IGHV7-3*01 F	Musmus IGHD2-3*01 F	Musmus IGHJ2*01 F	C104,F118	100	664	productive	2
**57**	A144E/WT	12 AA	ASGDSSGYPFDY	Musmus IGHV1-59*01 F	Musmus IGHD3-2*02 F	Musmus IGHJ2*01 F	C104,F118	100	598	productive	1
**58**	A144E/WT	12 AA	IRNNYVSGYFDY	Musmus IGHV6-6*01 F	Musmus IGHD1-1*01 F	Musmus IGHJ2*01 F	C104,F118	95.92	615	productive	2
**59**	A144E/WT	12 AA	TRGAEGNAFFDY	Musmus IGHV1-26*01 F	Musmus IGHD1-1*02 F	Musmus IGHJ2*01 F	C104,F118	94.79	579	productive	1
**60**	A144E/WT	12 AA	TRRDGHFEGFAY	Musmus IGHV14-4*01 F	Musmus IGHD2-3*01 F	Musmus IGHJ3*01 F	C104,F118	100	681	productive	1
**61**	A144E/WT	12 AA	VRLNWDVWYFDV	Musmus IGHV10-1*01 F	Musmus IGHD4-1*02 F	Musmus IGHJ1*03 F	C104,F118	100	376	productive	1
**62**	A144E/WT	11 AA	AKGGYGSPMDY	Musmus IGHV3-6*01 F	Musmus IGHD1-1*01 F	Musmus IGHJ4*01 F	C104,F118	100	587	productive	1
**63**	A144E/WT	11 AA	ARDARAGCFDY	Musmus IGHV7-1*01 F, or Musmus IGHV7-1*03 F	Musmus IGHD3-3*01 F	Musmus IGHJ2*01 F	C104,F118	100	603	productive	1
**64**	A144E/WT	11 AA	ARDGYGGAMDY	Musmus IGHV1-82*01 F	Musmus IGHD2-2*01 F	Musmus IGHJ4*01 F	C104,F118	99.23	408	productive	1
**65**	A144E/WT	11 AA	ARDLRAGAMDY	Musmus IGHV7-1*01 F, or Musmus IGHV7-1*03 F	Musmus IGHD1-1*01 F	Musmus IGHJ4*01 F	C104,F118	100	729	productive	24
**66**	A144E/WT	11 AA	AREELDRGFAY	Musmus IGHV3-6*01 F	Musmus IGHD3-2*01 F	Musmus IGHJ3*01 F	C104,F118	99.65	589	productive	1
**67**	A144E/WT	11 AA	AREHSNYYLDY	Musmus IGHV3-6*01 F	Musmus IGHD2-5*01 F	Musmus IGHJ2*01 F	C104,F118	96.88	588	productive	2
**68**	A144E/WT	11 AA	ARLGDYSWFAY	Musmus IGHV1-72*01 F	Musmus IGHD2-13*01 F	Musmus IGHJ3*01 F	C104,F118	100	381	productive	1
**69**	A144E/WT	11 AA	ARRGYFYAMDY	Musmus IGHV5-12*01 F	Musmus IGHD2-3*01 F	Musmus IGHJ4*01 F	C104,F118	99.65	622	productive	1
**70**	A144E/WT	11 AA	ARSGYYYDLLY	Musmus IGHV1-75*01 F	Musmus IGHD1-1*01 F, or Musmus IGHD1-1*02 F	Musmus IGHJ3*01 F	C104,F118	96.53	591	productive	2
**71**	A144E/WT	11 AA	ARTLWDRAMDY	Musmus IGHV2-2*01 F	Musmus IGHD4-1*01 F	Musmus IGHJ4*01 F	C104,F118	97.54	655	productive	1
**72**	A144E/WT	11 AA	ARTLYWDYFDY	Musmus IGHV2-2*01 F	Musmus IGHD2-3*01 F	Musmus IGHJ2*01 F, or Musmus IGHJ2*02 F	C104,F118	95.09	576	productive	1
**73**	A144E/WT	11 AA	ARVLRAGAMDY	Musmus IGHV7-1*01 F, or Musmus IGHV7-1*03 F	Musmus IGHD1-1*01 F	Musmus IGHJ4*01 F	C104,F118	96.26	493	unproductive	1
**74**	A144E/WT	11 AA	ARYITTEGFTY	Musmus IGHV7-3*01 F	Musmus IGHD1-1*02 F	Musmus IGHJ3*01 F	C104,F118	98.98	623	productive	3
**75**	A144E/WT	11 AA	ASIYYGYAMDY	Musmus IGHV3-6*01 F	Musmus IGHD1-1*01 F	Musmus IGHJ4*01 F	C104,F118	100	679	productive	4

**76**	A144E/WT	11 AA	MRYGNYWYFDV	Musmus IGHV11-2*01 F	Musmus IGHD2-1*01 F	Musmus IGHJ1*03 F	C104,F118	100	567	productive	1
**77**	A144E/WT	11 AA	MRYSNYWYFDV	Musmus IGHV11-2*01 F	Musmus IGHD2-5*01 F	Musmus IGHJ1*03 F	C104,F118	100	476	productive	1
**78**	A144E/WT	11 AA	TTPTLVSSFDY	Musmus IGHV14-1*01 F	Musmus IGHD1-1*01 F	Musmus IGHJ2*01 F	C104,F118	97.22	581	productive	1
**79**	A144E/WT	11 AA	VKGYEGGAMDY	Musmus IGHV9-1*01 F	Musmus IGHD2-2*01 F	Musmus IGHJ4*01 F	C104,F118	100	691	productive	1
**80**	A144E/WT	10 AA	AKGGDAWFAY	Musmus IGHV1-50*01 F	Musmus IGHD3-3*01 F	Musmus IGHJ3*01 F	C104,F118	100	659	productive	1
**81**	A144E/WT	10 AA	ARDGSYYFDY	Musmus IGHV3-6*01 F	Musmus IGHD3-1*01 F	Musmus IGHJ2*01 F	C104,F118	100	674	productive	1
**82**	A144E/WT	10 AA	ARHSYSNFDY	Musmus IGHV5-9*01 F	Musmus IGHD2-5*01 F	Musmus IGHJ2*01 F	C104,F118	100	685	productive	1
**83**	A144E/WT	10 AA	ARLAGGEEDY	Musmus IGHV1-50*01 F	Musmus IGHD3-3*01 F	Musmus IGHJ2*01 F	C104,F118	100	659	productive	1
**84**	A144E/WT	10 AA	ARLTGTSFAY	Musmus IGHV5-17*01 F	Musmus IGHD4-1*01 F	Musmus IGHJ3*01 F	C104,F118	100	584	productive	1
**85**	A144E/WT	10 AA	ASALFGPLAF	Musmus IGHV1-18*01 F	Musmus IGHD6-2*01 F	Musmus IGHJ3*01 F	C104,F118	96.53	586	productive	2
**86**	A144E/WT	10 AA	MRYGGFYFDY	Musmus IGHV11-2*01 F	Musmus IGHD1-1*02 F	Musmus IGHJ2*01 F	C104,F118	98.61	583	productive	1
**87**	A144E/WT	10 AA	TIGYYYAMDY	Musmus IGHV1-74*01 F	Musmus IGHD2-2*01 F	Musmus IGHJ4*01 F	C104,F118	99.31	588	productive	1
**88**	A144E/WT	10 AA	TRDVNVYFDY	Musmus IGHV1-15*01 F	Musmus IGHD2-1*01 F	Musmus IGHJ2*01 F	C104,F118	99.31	545	productive	1
**89**	A144E/WT	10 AA	TRPVSSHFDY	Musmus IGHV6-6*01 F	Musmus IGHD2-12*01 F	Musmus IGHJ2*01 F	C104,F118	99.32	697	productive	1
**90**	A144E/WT	10 AA	TSALFGPLAY	Musmus IGHV1-18*01 F	Musmus IGHD6-2*01 F	Musmus IGHJ3*01 F	C104,F118	96.88	589	productive	1
**91**	A144E/WT	10 AA	VRDTGEWFAY	Musmus IGHV10-3*01 F	Musmus IGHD4-1*01 F	Musmus IGHJ3*01 F	C104,F118	99.32	602	productive	1
**92**	A144E/WT	9 AA	AIGWDEFDY	Musmus IGHV1-74*01 F	Musmus IGHD1-2*01 F	Musmus IGHJ2*01 F	C104,F118	96.88	590	productive	2
**93**	A144E/WT	9 AA	ANYYGSFDY	Musmus IGHV1-78*01 F	Musmus IGHD1-1*01 F	Musmus IGHJ2*01 F	C104,F118	100	552	productive	1
**94**	A144E/WT	9 AA	ARDYDGFAY	Musmus IGHV1-7*01 F	Musmus IGHD2-4*01 F	Musmus IGHJ3*01 F	C104,F118	97.57	523	productive	1
**95**	A144E/WT	9 AA	ARGGNYFDY	Musmus IGHV1-26*01 F	Musmus IGHD1-1*02 F	Musmus IGHJ2*01 F	C104,F118	100	672	productive	1
**96**	A144E/WT	9 AA	ARLGEAMDY	Musmus IGHV1-75*01 F	Musmus IGHD1-1*02 F	Musmus IGHJ4*01 F	C104,F118	100	573	productive	1
**97**	A144E/WT	9 AA	ARTAGSFAY	Musmus IGHV1-9*01 F	Musmus IGHD3-1*01 F	Musmus IGHJ3*01 F	C104,F118	100	673	productive	1
**98**	A144E/WT	9 AA	ARYGGGFAY	Musmus IGHV1-54*01 F	Musmus IGHD1-1*02 F	Musmus IGHJ3*01 F	C104,F118	99.65	583	productive	1
**99**	A144E/WT	9 AA	TGRWGYFDV	Musmus IGHV1-5*01 F	Musmus IGHD3-3*01 F	Musmus IGHJ1*03 F	C104,F118	99.31	571	productive	2
**100**	A144E/WT	8 AA	APWAAMDY	Musmus IGHV1-26*01 F	Musmus IGHD4-1*01 F	Musmus IGHJ4*01 F	C104,F118	97.57	579	productive	1
**101**	A144E/WT	8 AA	ARSYDFDY	Musmus IGHV5-17*01 F	Musmus IGHD1-1*01 F	Musmus IGHJ2*01 F	C104,F118	100	668	productive	1
**102**	A144E/WT	8 AA	ARVRAMDY	Musmus IGHV1-53*01 F	Musmus IGHD2-13*01 F	Musmus IGHJ4*01 F	C104,F118	100	673	productive	1

**103**	A144E/WT	8 AA	ARYWSFDF	Musmus IGHV9-4*01 F	Musmus IGHD4-1*01 F	Musmus IGHJ2*01 F	C104,F118	98.61	680	productive	1
**104**	A144E/WT	8 AA	TDGRNFDV	Musmus IGHV6-6*01 F	Musmus IGHD1-1*01 F	Musmus IGHJ1*03 F	C104,F118	96.94	608	productive	1
**105**	A144E/WT	8 AA	VNWDAMDY	Musmus IGHV10-1*01 F	Musmus IGHD4-1*01 F	Musmus IGHJ4*01 F	C104,F118	100	686	productive	1
**106**	A144E/WT	7 AA	AKQAMDY	Musmus IGHV2-3*01 F		Musmus IGHJ4*01 F	C104,F118	100	643	productive	1
**107**	A144E/WT	7 AA	TRNPLDY	Musmus IGHV14-4*01 F	Musmus IGHD2-1*01 F	Musmus IGHJ2*01 F	C104,F118	100	661	productive	1

Table S6: Identity and query names of the Immunoglobulin Light chain clones from A144E/WT or A144E/A144E IgA-positive *C. rodentium*-bound B cells sorted from Peyer's patches 14 days after infection.

	Genotype	CDR3-IMGT length (AA)	CDR3-IMGT sequence (AA)	V gene and allele	D gene and allele	J gene and allele	Anchors 104,118	V %	Seq. length	Function	
1	A144E/A144E	10 AA	SQSTHVPPYT	Musmus IGKV1-110*01 F	NA	Musmus IGKJ2*01 F	C104,F118	100	531	productive	1
2	A144E/A144E	9 AA	LQSDNMPLT	Musmus IGKV17-127*01 F	NA	Musmus IGKJ5*01 F	C104,F118	98.57	499	productive	1
3	A144E/A144E	9 AA	LQYSSSPFT	Musmus IGKV9-120*01 F	NA	Musmus IGKJ4*01 F	C104,F118	98.57	501	productive	1
4	A144E/A144E	9 AA	MQHLETPYT	Musmus IGKV2-137*01 F	NA	Musmus IGKJ2*01 F, or Musmus IGKJ2*02 F	C104,F118	97.96	510	productive	1
5	A144E/A144E	9 AA	QQFNNYPYT	Musmus IGKV6-15*01 F	NA	Musmus IGKJ2*01 F	C104,F118	98.21	501	productive	2
6	A144E/A144E	9 AA	QQFNSYPYT	Musmus IGKV6-15*01 F	NA	Musmus IGKJ2*01 F	C104,F118	98.92	534	productive	2
7	A144E/A144E	9 AA	QQGNTLPYT	Musmus IGKV10-96*01 F	NA	Musmus IGKJ2*01 F	C104,F118	98.21	500	productive	1
8	A144E/A144E	9 AA	QQGSTIPRT	Musmus IGKV4-91*01 F	NA	Musmus IGKJ4*01 F, or Musmus IGKJ4*02 F	C104,F118	97.52	508	productive	1
9	A144E/A144E	9 AA	QQSNDWPYT	Musmus IGKV5-48*01 F	NA	Musmus IGKJ2*01 F	C104,F118	97.85	505	productive	1
10	A144E/A144E	9 AA	QQYNSYPFT	Musmus IGKV6-15*01 F	NA	Musmus IGKJ2*01 F	C104,F118	97.85	516	productive	1
11	A144E/A144E	9 AA	QQYSSYPYT	Musmus IGKV6-23*01 F	NA	Musmus IGKJ2*01 F	C104,F118	100	500	productive	1
12	A144E/A144E	8 AA	FQGSHVRT	Musmus IGKV1-117*01 F	NA	Musmus IGKJ1*01 F	C104,F118	100	519	productive	1

	Genotype	CDR3-IMGT length (AA)	CDR3-IMGT sequence (AA)	V gene and allele	D gene and allele	J gene and allele	Anchors 104,118	V %	Seque nce length	Function	#
		FQGSHVP YT									
1	A144E/A144E	9 AA	FQGSHVPYT	Musmus IGKV1-117*01 F	NA	Musmus IGKJ2*01 F	C104,F118	100	522	productive	1
	A144E/WT	9 AA	FQGSHVPYT	Musmus IGKV1-117*01 F	NA	Musmus IGKJ2*01 F	C104,F118	100	522	productive	7
		LQYASSPWT									
2	A144E/A144E	9 AA	LQYASSPWT	Musmus IGKV9-120*01 F	NA	Musmus IGKJ1*01 F	C104,F118	99.64	536	productive	1
	A144E/WT	9 AA	LQYASSPWT	Musmus IGKV9-120*01 F	NA	Musmus IGKJ1*01 F	C104,F118	99.64	501	productive	4
		QHHYGTPYT									

3	A144E/A144E	9 AA	QHHYGTPYT	Musmus IGKV12-44*01 F	NA	Musmus IGKJ2*01 F	C104,F118	99.64	499	productive	1
	A144E/WT	9 AA	QHHYGTPYT	Musmus IGKV12-44*01 F	NA	Musmus IGKJ2*01 F	C104,F118	95.7	499	productive	2
		SQSTHVPYT									
4	A144E/A144E	9 AA	SQSTHVPYT	Musmus IGKV1-110*01 F	NA	Musmus IGKJ2*01 F	C104,F118	97.96	535	productive	1
	A144E/WT	9 AA	SQSTHVPYT	Musmus IGKV1-110*01 F	NA	Musmus IGKJ2*01 F	C104,F118	100	563	productive	1

	Genotype	CDR3-IMGT length (AA)	CDR3-IMGT sequence (AA)	V gene and allele	D gene and allele	J gene and allele	Anchors 104,118	V %	Sequence length	Function	#
1	A144E/WT	10 AA	LQYDNLLQYT	Musmus IGKV19-93*01 F	NA	Musmus IGKJ2*01 F	C104,F118	100	371	productive	1
2	A144E/WT	10 AA	QQGSSIPRIT	Musmus IGKV4-91*01 F	NA	Musmus IGKJ4*01 F	C104,F118	100	497	productive	1
3	A144E/WT	9 AA	AQNLELPWT	Musmus IGKV2-109*01 F	NA	Musmus IGKJ1*01 F	C104,F118	100	515	productive	1
4	A144E/WT	9 AA	FQGSHVPFT	Musmus IGKV1-117*01 F	NA	Musmus IGKJ4*01 F	C104,F118	100	522	productive	1
5	A144E/WT	9 AA	FQGSHVPPT	Musmus IGKV1-117*01 F	NA	Musmus IGKJ5*01 F	C104,F118	97.96	522	productive	2
6	A144E/WT	9 AA	FQGSHVPPT	Musmus IGKV1-117*01 F	NA	Musmus IGKJ1*01 F	C104,F118	99.66	522	productive	1
7	A144E/WT	9 AA	FQGSHVPRT	Musmus IGKV1-117*01 F	NA	Musmus IGKJ1*01 F	C104,F118	99.66	522	productive	1
8	A144E/WT	9 AA	FQGSHVPWT	Musmus IGKV1-117*01 F	NA	Musmus IGKJ1*01 F	C104,F118	100	521	productive	1
9	A144E/WT	9 AA	GQSYSYPLT	Musmus IGKV14-126*01 F	NA	Musmus IGKJ5*01 F	C104,F118	90.98	369	productive	1
10	A144E/WT	9 AA	GQSYSYPYT	Musmus IGKV6-20*01 F	NA	Musmus IGKJ2*01 F	C104,F118	100	493	productive	1
11	A144E/WT	9 AA	HQWSSYPWT	Musmus IGKV4-80*01 F	NA	Musmus IGKJ1*01 F	C104,F118	100	493	productive	1
12	A144E/WT	9 AA	KQSYNLPLT	Musmus IGKV8-21*01 F	NA	Musmus IGKJ5*01 F	C104,F118	100	503	productive	5
13	A144E/WT	9 AA	LQGTHQPRT	Musmus IGKV1-88*01 F	NA	Musmus IGKJ1*01 F	C104,F118	98.64	438	productive	1
14	A144E/WT	9 AA	LQHGESPFT	Musmus IGKV14-126*01 F	NA	Musmus IGKJ4*01 F	C104,F118	100	507	productive	3
15	A144E/WT	9 AA	LQHGESPWT	Musmus IGKV14-126*01 F	NA	Musmus IGKJ1*01 F	C104,F118	100	497	productive	1
16	A144E/WT	9 AA	LQHGESPYT	Musmus IGKV14-126*01 F	NA	Musmus IGKJ2*01 F	C104,F118	100	508	productive	36
17	A144E/WT	9 AA	LQRNVFPYT	Musmus IGKV9-123*01 F	NA	Musmus IGKJ2*01 F	C104,F118	98.92	495	productive	1
18	A144E/WT	9 AA	LQSDNLPLT	Musmus IGKV17-121*01 F	NA	Musmus IGKJ5*01 F	C104,F118	100	501	productive	2
19	A144E/WT	9 AA	LQSDNLPYT	Musmus IGKV17-121*01 F	NA	Musmus IGKJ2*01 F	C104,F118	100	493	productive	1
20	A144E/WT	9 AA	LQSDNMPYT	Musmus IGKV17-127*01 F	NA	Musmus IGKJ2*01 F	C104,F118	100	502	productive	6
21	A144E/WT	9 AA	LQVTHVPRT	Musmus IGKV1-122*01 F	NA	Musmus IGKJ1*01 F	C104,F118	100	501	productive	1
22	A144E/WT	9 AA	LQYASSPYT	Musmus IGKV9-120*01 F	NA	Musmus IGKJ2*01 F	C104,F118	99.64	494	productive	2

23	A144E/WT	9 AA	LQYDEFPYT	Musmus IGKV14-111*01 F	NA	Musmus IGKJ2*01 F	C104,F118	100	501	productive	1
24	A144E/WT	9 AA	MQHLEYPYT	Musmus IGKV2-137*01 F	NA	Musmus IGKJ2*01 F	C104,F118	100	517	productive	1
25	A144E/WT	9 AA	PQHGESPFT	Musmus IGKV14-126*01 F	NA	Musmus IGKJ4*01 F	C104,F118	99.06	338	productive	1
26	A144E/WT	9 AA	Q*WSGYPLT	Musmus IGKV4-58*01 F	NA	Musmus IGKJ5*01 F	C104,F118	96.81	615	unproductiv e	1
27	A144E/WT	9 AA	QHFWSTPLT	Musmus IGKV12-41*01 F	NA	Musmus IGKJ5*01 F	C104,F118	100	494	productive	1
28	A144E/WT	9 AA	QHHYGIPYT	Musmus IGKV12-44*01 F	NA	Musmus IGKJ2*01 F	C104,F118	96.42	499	productive	8
29	A144E/WT	9 AA	QHHYGPPYT	Musmus IGKV12-44*01 F	NA	Musmus IGKJ2*01 F	C104,F118	94.62	496	productive	1
30	A144E/WT	9 AA	QHHYGTPFT	Musmus IGKV12-44*01 F	NA	Musmus IGKJ4*01 F	C104,F118	100	508	productive	2
31	A144E/WT	9 AA	QHSRELPYT	Musmus IGKV3-12*01 F	NA	Musmus IGKJ2*01 F	C104,F118	100	580	productive	2
32	A144E/WT	9 AA	QHSWEIPYT	Musmus IGKV3-7*01 F	NA	Musmus IGKJ2*01 F	C104,F118	99.66	505	productive	1
33	A144E/WT	9 AA	QHTWEIPYT	Musmus IGKV3-7*01 F	NA	Musmus IGKJ2*01 F	C104,F118	97.25	505	productive	1
34	A144E/WT	9 AA	QHYYGTPYT	Musmus IGKV12-40*01 P	NA	Musmus IGKJ2*01 F	C104,F118	100	490	unproductiv e	1
35	A144E/WT	9 AA	QNDHRYPYT	Musmus IGKV8-28*01 F	NA	Musmus IGKJ2*01 F	C104,F118	97.98	517	productive	1
36	A144E/WT	9 AA	QNDYSYPLT	Musmus IGKV8-19*01 F	NA	Musmus IGKJ5*01 F	C104,F118	100	511	productive	1
37	A144E/WT	9 AA	QNGHSFPLT	Musmus IGKV5-39*01 F	NA	Musmus IGKJ5*01 F	C104,F118	100	623	productive	1
38	A144E/WT	9 AA	QNSWEIPYT	Musmus IGKV3-7*01 F	NA	Musmus IGKJ2*01 F	C104,F118	95.19	528	productive	1 7
39	A144E/WT	9 AA	QNVLSTPFT	Musmus IGKV12-89*01 F	NA	Musmus IGKJ4*01 F	C104,F118	100	512	productive	4
40	A144E/WT	9 AA	QNVLSTPPT	Musmus IGKV12-89*01 F	NA	Musmus IGKJ1*01 F	C104,F118	100	491	productive	2
41	A144E/WT	9 AA	QNVLSTPRT	Musmus IGKV12-89*01 F	NA	Musmus IGKJ1*01 F	C104,F118	100	504	productive	3
42	A144E/WT	9 AA	QNVLSTPWT	Musmus IGKV12-89*01 F	NA	Musmus IGKJ1*01 F	C104,F118	100	505	productive	6
43	A144E/WT	9 AA	QNVLSTPYT	Musmus IGKV12-89*01 F	NA	Musmus IGKJ2*01 F	C104,F118	100	508	productive	1
44	A144E/WT	9 AA	QQDYSSPPT	Musmus IGKV6-32*01 F, or Musmus IGKV6-32*02 F	NA	Musmus IGKJ5*01 F	C104,F118	98.56	335	productive	1
45	A144E/WT	9 AA	QQGDALPRT	Musmus IGKV10-96*01 F	NA	Musmus IGKJ1*01 F	C104,F118	97.85	501	productive	3
46	A144E/WT	9 AA	QQGHTLPWT	Musmus IGKV10-96*01 F	NA	Musmus IGKJ1*01 F	C104,F118	98.92	500	productive	2
47	A144E/WT	9 AA	QQGNALPRT	Musmus IGKV10-96*01 F	NA	Musmus IGKJ1*01 F	C104,F118	98.92	502	productive	9
48	A144E/WT	9 AA	QQGNTLPFT	Musmus IGKV10-96*01 F	NA	Musmus IGKJ4*01 F	C104,F118	99.64	537	productive	2
49	A144E/WT	9 AA	QQGNTLPRT	Musmus IGKV10-96*01 F	NA	Musmus IGKJ1*01 F	C104,F118	100	510	productive	1
50	A144E/WT	9 AA	QQGNTLPWT	Musmus IGKV10-96*01 F	NA	Musmus IGKJ1*01 F	C104,F118	100	503	productive	3
51	A144E/WT	9 AA	QQGQSYPWT	Musmus IGKV15-103*01 ORF	NA	Musmus IGKJ1*01 F	C104,F118	100	493	productive	1

52	A144E/WT	9 AA	QQGSSIPFT	Musmus IGKV4-91*01 F	NA	Musmus IGKJ4*01 F	C104,F118	100	524	productive	5
53	A144E/WT	9 AA	QQGSSIPLT	Musmus IGKV4-91*01 F	NA	Musmus IGKJ5*01 F	C104,F118	100	585	productive	6
54	A144E/WT	9 AA	QQGSSIPRT	Musmus IGKV4-91*01 F	NA	Musmus IGKJ4*01 F	C104,F118	100	492	productive	1
55	A144E/WT	9 AA	QQGSSIPRT	Musmus IGKV4-91*01 F	NA	Musmus IGKJ2*01 F	C104,F118	100	585	productive	1
56	A144E/WT	9 AA	QQHFTTPFT	Musmus IGKV6-25*01 F	NA	Musmus IGKJ2*01 F	C104,F118	95.7	498	productive	1
57	A144E/WT	9 AA	QQHLHIPLT	Musmus IGKV8-16*01 F	NA	Musmus IGKJ5*01 F	C104,F118	100	329	productive	1
58	A144E/WT	9 AA	QQHNEYPLT	Musmus IGKV16-104*01 F	NA	Musmus IGKJ5*01 F	C104,F118	99.64	495	productive	1
59	A144E/WT	9 AA	QQHYSIPYT	Musmus IGKV6-25*01 F	NA	Musmus IGKJ2*01 F	C104,F118	97.85	498	productive	2
60	A144E/WT	9 AA	QQHYSTPLT	Musmus IGKV8-24*01 F	NA	Musmus IGKJ5*01 F	C104,F118	100	512	productive	1
61	A144E/WT	9 AA	QQHYSTPRT	Musmus IGKV6-17*01 F	NA	Musmus IGKJ1*01 F	C104,F118	99.28	495	productive	1
62	A144E/WT	9 AA	QQHYSTPWT	Musmus IGKV6-25*01 F	NA	Musmus IGKJ1*01 F	C104,F118	100	375	productive	1
63	A144E/WT	9 AA	QQHYTIPYT	Musmus IGKV6-25*01 F	NA	Musmus IGKJ2*01 F	C104,F118	97.13	498	productive	2
64	A144E/WT	9 AA	QQNNEDPFT	Musmus IGKV3-10*01 F	NA	Musmus IGKJ4*01 F	C104,F118	100	532	productive	2
65	A144E/WT	9 AA	QQRSSYPLT	Musmus IGKV4-57*01 F	NA	Musmus IGKJ5*01 F	C104,F118	100	555	productive	3
66	A144E/WT	9 AA	QQRSSYPYT	Musmus IGKV4-90*01 F	NA	Musmus IGKJ2*01 F	C104,F118	100	504	productive	1
67	A144E/WT	9 AA	QQRSTYPRT	Musmus IGKV4-57*01 F	NA	Musmus IGKJ1*01 F	C104,F118	96.74	544	productive	1
68	A144E/WT	9 AA	QQSKEVPFT	Musmus IGKV3-2*01 F	NA	Musmus IGKJ4*01 F	C104,F118	99.66	496	productive	1
69	A144E/WT	9 AA	QQSKEVPYT	Musmus IGKV3-2*01 F	NA	Musmus IGKJ2*01 F	C104,F118	100	496	productive	1
70	A144E/WT	9 AA	QQSNEDPRT	Musmus IGKV3-4*01 F	NA	Musmus IGKJ1*01 F	C104,F118	99.66	523	productive	1
71	A144E/WT	9 AA	QQSNEDPYT	Musmus IGKV3-4*01 F	NA	Musmus IGKJ2*01 F	C104,F118	100	527	productive	3
72	A144E/WT	9 AA	QQSNSWPFT	Musmus IGKV5-43*01 F	NA	Musmus IGKJ4*01 F	C104,F118	100	497	productive	2
73	A144E/WT	9 AA	QQSNSWPLT	Musmus IGKV5-43*01 F	NA	Musmus IGKJ5*01 F	C104,F118	100	497	productive	3<br>5
74	A144E/WT	9 AA	QQSNSWPTT	Musmus IGKV5-48*01 F	NA	Musmus IGKJ1*01 F	C104,F118	100	498	productive	1
75	A144E/WT	9 AA	QQSNSWPYT	Musmus IGKV5-48*01 F	NA	Musmus IGKJ2*01 F	C104,F118	100	502	productive	1
76	A144E/WT	9 AA	QQSSSWPFT	Musmus IGKV5-48*01 F	NA	Musmus IGKJ2*01 F	C104,F118	98.92	502	productive	1
77	A144E/WT	9 AA	QQTNSWPFT	Musmus IGKV5-45*01 F	NA	Musmus IGKJ5*01 F	C104,F118	98.21	503	productive	1
78	A144E/WT	9 AA	QQWSDYPLT	Musmus IGKV4-58*01 F	NA	Musmus IGKJ5*01 F	C104,F118	97.87	528	productive	1
79	A144E/WT	9 AA	QQWSGNPLT	Musmus IGKV4-72*01 F	NA	Musmus IGKJ5*01 F	C104,F118	96.38	477	productive	2
80	A144E/WT	9 AA	QQWSGYPLT	Musmus IGKV4-58*01 F	NA	Musmus IGKJ5*01 F	C104,F118	99.65	476	productive	9

81	A144E/WT	9 AA	QQWSSNPLT	Musmus IGKV4-59*01 F	NA	Musmus IGKJ5*01 F	C104,F118	100	494	productive	3
82	A144E/WT	9 AA	QQWSSNPLT	Musmus IGKV4-72*01 F	NA	Musmus IGKJ5*01 F	C104,F118	100	363	productive	1
83	A144E/WT	9 AA	QQWSSNPVT	Musmus IGKV4-59*01 F	NA	Musmus IGKJ2*01 F	C104,F118	100	494	productive	1
84	A144E/WT	9 AA	QQWSSNPYT	Musmus IGKV4-68*01 F	NA	Musmus IGKJ2*01 F	C104,F118	100	521	productive	2
85	A144E/WT	9 AA	QQWSSNPYT	Musmus IGKV4-59*01 F	NA	Musmus IGKJ2*01 F	C104,F118	100	504	productive	1
86	A144E/WT	9 AA	QQWSSYPFT	Musmus IGKV4-53*01 F	NA	Musmus IGKJ4*01 F	C104,F118	100	651	productive	1
87	A144E/WT	9 AA	QQWSSYPYT	Musmus IGKV4-55*01 F	NA	Musmus IGKJ2*01 F	C104,F118	100	490	productive	1
88	A144E/WT	9 AA	QQYNSYPLT	Musmus IGKV6-15*01 F	NA	Musmus IGKJ5*01 F	C104,F118	100	501	productive	1
89	A144E/WT	9 AA	QQYNSYPLT	Musmus IGKV6-15*01 F	NA	Musmus IGKJ1*01 F	C104,F118	100	496	productive	1
90	A144E/WT	9 AA	QQYNSYPLT	Musmus IGKV6-15*01 F	NA	Musmus IGKJ4*01 F	C104,F118	100	498	productive	1
91	A144E/WT	9 AA	QQYSGYPLT	Musmus IGKV4-57-1*01 F	NA	Musmus IGKJ2*01 F	C104,F118	100	544	productive	1
92	A144E/WT	9 AA	QQYSGYPLT	Musmus IGKV4-57-1*01 F	NA	Musmus IGKJ5*01 F	C104,F118	100	528	productive	1
93	A144E/WT	9 AA	QQYSKLPFT	Musmus IGKV10-94*01 F	NA	Musmus IGKJ4*01 F	C104,F118	99.64	623	productive	1
94	A144E/WT	9 AA	QQYSSYPFT	Musmus IGKV6-23*01 F	NA	Musmus IGKJ4*01 F	C104,F118	100	496	productive	1
95	A144E/WT	9 AA	QQYTKLPWT	Musmus IGKV10-94*01 F	NA	Musmus IGKJ1*01 F	C104,F118	97.49	501	productive	1
96	A144E/WT	9 AA	QQYYSYPFT	Musmus IGKV8-30*01 F	NA	Musmus IGKJ4*01 F	C104,F118	100	509	productive	4
97	A144E/WT	9 AA	QQYYSYPLT	Musmus IGKV8-30*01 F	NA	Musmus IGKJ5*01 F	C104,F118	100	507	productive	2
98	A144E/WT	9 AA	QQYYSYPWT	Musmus IGKV8-30*01 F	NA	Musmus IGKJ1*01 F	C104,F118	100	505	productive	1
99	A144E/WT	9 AA	QQYYSYPYT	Musmus IGKV8-30*01 F	NA	Musmus IGKJ2*01 F	C104,F118	100	515	productive	1
100	A144E/WT	9 AA	SQSTHVPFT	Musmus IGKV1-110*01 F	NA	Musmus IGKJ4*01 F	C104,F118	100	527	productive	3
101	A144E/WT	9 AA	SQSTHVPPT	Musmus IGKV1-110*01 F	NA	Musmus IGKJ4*01 F	C104,F118	99.32	526	productive	1
102	A144E/WT	9 AA	SQSTHVPRT	Musmus IGKV1-110*01 F	NA	Musmus IGKJ1*01 F	C104,F118	100	525	productive	3
103	A144E/WT	9 AA	SQSTHVPWT	Musmus IGKV1-110*01 F	NA	Musmus IGKJ1*01 F	C104,F118	100	524	productive	4
104	A144E/WT	9 AA	SQTTHVPYT	Musmus IGKV1-110*01 F	NA	Musmus IGKJ2*01 F	C104,F118	96.94	562	productive	4
105	A144E/WT	9 AA	VQFAQFPWT	Musmus IGKV14-100*01 F	NA	Musmus IGKJ1*01 F	C104,F118	98.92	528	productive	1
106	A144E/WT	9 AA	VQYAHFPWT	Musmus IGKV14-100*01 F	NA	Musmus IGKJ1*01 F	C104,F118	98.57	504	productive	4
107	A144E/WT	9 AA	WQGTHFPFT	Musmus IGKV1-135*01 F	NA	Musmus IGKJ4*01 F	C104,F118	100	512	productive	1
108	A144E/WT	9 AA	WQGTHFPHT	Musmus IGKV1-135*01 F	NA	Musmus IGKJ2*01 F	C104,F118	100	511	productive	2
109	A144E/WT	9 AA	WQGTHFPLT	Musmus IGKV1-135*01 F	NA	Musmus IGKJ5*01 F	C104,F118	100	512	productive	1

110	A144E/WT	8 AA	HQRSSYYT	Musmus IGKV4-70*01 F	NA	Musmus IGKJ2*01 F	C104,F118	100	534	productive	1
111	A144E/WT	8 AA	HQWSSYRT	Musmus IGKV4-80*01 F	NA	Musmus IGKJ1*01 F	C104,F118	100	490	productive	1
112	A144E/WT	8 AA	HQYLSSLT	Musmus IGKV8-27*01 F	NA	Musmus IGKJ5*01 F	C104,F118	100	511	productive	1
113	A144E/WT	8 AA	HQYLSSRT	Musmus IGKV8-27*01 F	NA	Musmus IGKJ1*01 F	C104,F118	100	511	productive	1
114	A144E/WT	8 AA	KQSYNLRT	Musmus IGKV8-21*01 F	NA	Musmus IGKJ1*01 F	C104,F118	98.65	499	productive	1
115	A144E/WT	8 AA	LQSDNMLT	Musmus IGKV17-127*01 F	NA	Musmus IGKJ5*01 F	C104,F118	100	491	productive	1
116	A144E/WT	8 AA	LQYDELYT	Musmus IGKV14-111*01 F	NA	Musmus IGKJ2*01 F	C104,F118	100	496	productive	1
117	A144E/WT	8 AA	QQGSSMLT	Musmus IGKV4-91*01 F	NA	Musmus IGKJ5*01 F	C104,F118	100	500	productive	2
118	A144E/WT	8 AA	QQYYNYRT	Musmus IGKV8-30*01 F	NA	Musmus IGKJ1*01 F	C104,F118	97.64	527	productive	1
119	A144E/WT	8 AA	QQYYSYRT	Musmus IGKV8-30*01 F	NA	Musmus IGKJ1*01 F	C104,F118	98.32	505	productive	1
120	A144E/WT	7 AA	HQRSSLT	Musmus IGKV4-69*01 F	NA	Musmus IGKJ5*01 F	C104,F118	97.89	441	productive	1
	**Genotype**	**CDR3-IMGT length (AA)**	**CDR3-IMGT sequence (AA)**	**V gene and allele**	**D gene and allele**	**J gene and allele**	**Anchors 104,118**	**V %**	**Length**	**Function**	**#**
1	A144E/WT	13 AA	GVGDTIKEQFVYV	Musmus IGLV3*01 F	NA	Musmus IGLJ2*01 F	C104,F118	99.66	559	productive	1
2	A144E/WT	10 AA	ALWYSNHFWV	Musmus IGLV1*01 F	NA	Musmus IGLJ1*01 F	C104,F118	100	401	productive	2
3	A144E/WT	9 AA	ALWYSNHFI	Musmus IGLV1*01 F	NA	Musmus IGLJ3*01 F	C104,F118	98.26	549	productive	2
4	A144E/WT	9 AA	ALWYSNHFV	Musmus IGLV1*01 F	NA	Musmus IGLJ1*01 F	C104,F118	98.96	509	productive	2
5	A144E/WT	9 AA	ALWYSNHLV	Musmus IGLV1*01 F	NA	Musmus IGLJ1*01 F	C104,F118	100	506	productive	1
6	A144E/WT	9 AA	ALWYSNHWV	Musmus IGLV1*01 F	NA	Musmus IGLJ1*01 F	C104,F118	100	509	productive	34
7	A144E/WT	9 AA	ALWYSNLWV	Musmus IGLV2*02 F	NA	Musmus IGLJ1*01 F	C104,F118	98.96	507	productive	1
8	A144E/WT	9 AA	ALWYSNQFI	Musmus IGLV1*01 F	NA	Musmus IGLJ3*01 F	C104,F118	100	546	productive	1
9	A144E/WT	9 AA	ALWYSTHFI	Musmus IGLV2*02 F	NA	Musmus IGLJ3*01 F	C104,F118	99.65	545	productive	1
10	A144E/WT	9 AA	ALWYSTHFV	Musmus IGLV2*02 F	NA	Musmus IGLJ2*01 F	C104,F118	100	581	productive	9
11	A144E/WT	9 AA	ALWYSTHWV	Musmus IGLV2*02 F	NA	Musmus IGLJ1*01 F	C104,F118	100	510	productive	1
12	A144E/WT	9 AA	ALWYSTHYV	Musmus IGLV2*02 F	NA	Musmus IGLJ2*01 F	C104,F118	100	565	productive	5
13	A144E/WT	9 AA	TLWYSNHWV	Musmus IGLV1*01 F	NA	Musmus IGLJ1*01 F	C104,F118	98.26	509	productive	1
14	A144E/WT	9 AA	VLWYSNIWV	Musmus IGLV1*01 F	NA	Musmus IGLJ1*01 F	C104,F118	98.26	509	productive	1

Table S7. Primers used for immunoglobulin genes amplification and sequencing. Restriction sites are underlined. [a]Forward primers used in 2nd PCRs were used for sequencing.

PCR step	Name	5′–3′ sequence
Igh 1st PCR	5′ MsVHE	GGGAATTCGAGGTGCAGCTGCAGGAGT CTGG
	3′ Cμ outer	AGGGGGCTCTCGCAGGAGACGAGG
	3′ Cγ1 outer	GGAAGGTGTGCACACCGCTGGAC
	3′ Cγ2c outer	GGAAGGTGTGCACACCACTGGAC
	3′ Cγ2b outer	GGAAGGTGTGCACACTGCTGGAC
	3′ Cγ3 outer	AGACTGTGCGCACACCGCTGGAC
	3′ Cα outer	GAAAGTTCACGGTGGTTATATCC
Igh 2nd PCR[a]	5′ MsVHE	GGGAATTCGAGGTGCAGCTGCAGGAGT CTGG
	3′ Cμ inner	AGGGGGAAGACATTTGGGAAGGAC
	3′ Cγ1 inner	GCTCAGGGAAATAGCCCTTGAC
	3′ Cγ2c inner	GCTCAGGGAAATAACCCTTGAC
	3′ Cγ2b inner	ACTCAGGGAAGTAGCCCTTGAC
	3′ Cγ3 inner	GCTCAGGGAAGTAGCCTTTGAC
	3′ Cα inner	TGCCGAAAGGGAAGTAATCGTGAAT
Igk 1st PCR	5′ L-Vκ_3	TGCTGCTGCTCTGGGTTCCAG
	5′ L-Vκ_4	ATTWTCAGCTTCCTGCTAATC
	5′ L-Vκ_5	TTTTGCTTTTCTGGATTYCAG
	5′ L-Vκ_6	TCGTGTTKCTSTGGTTGTCTG
	5′ L-Vκ_6,8,9	ATGGAATCACAGRCYCWGGT
	5′ L-Vκ_14	TCTTGTTGCTCTGGTTYCCAG
	5′ L-Vκ_19	CAGTTCCTGGGGCTCTTGTTGTTC
	5′ L-Vκ_20	CTCACTAGCTCTTCTCCTC
	3′ mCκ	GATGGTGGGAAGATGGATACAGTT
Igk 2nd PCR[a]	5′ mVkappa	GAYATTGTGMTSACMCARWCTMCA
	3′ BsiWI P-mJK01	GCCACCGTACGTTTGATTTCCAGCTTGG TG
	3′ BsiWI P-mJK02	GCCACCGTACGTTTTATTTCCAGCTTGG TC

PCR step	Name	5′–3′ sequence
	3′ BsiWI P-mJK03	GCCACCGTACGTTTTATTTCCAACTTTGTC
	3′ BsiWI P-mJK04	GCCACCGTACGTTTCAGCTCCAGCTTGGTC
Igl 1st PCR	5′ mVλ1/2	CAGGCTGTTGTGACTCAG
	5′ mVλx	CAACTTGTGCTCACTCAG
	3′ mCλ outer	GTACCATYTGCCTTCCAGKCCACT
Igl 2nd PCR[a]	5′ mVλ1/2	CAGGCTGTTGTGACTCAG
	5′ mVλx	CAACTTGTGCTCACTCAG
	3′ mCλ inner	CTCYTCAGRGGAAGGTGGRAACA

Table S8. Barcoded dual-index primers used for 16S RNA amplification and sequencing.

Name	5′–3′ sequence
v4.SA501	AATGATACGGCGACCACCGAGATCTACACATCGTACGTATGGTAATTGTGTGCCAGCMGCCGCGGTAA
v4.SA502	AATGATACGGCGACCACCGAGATCTACACACTATCTGTATGGTAATTGTGTGCCAGCMGCCGCGGTAA
v4.SA503	AATGATACGGCGACCACCGAGATCTACACTAGCGAGTTATGGTAATTGTGTGCCAGCMGCCGCGGTAA
v4.SA504	AATGATACGGCGACCACCGAGATCTACACCTGCGTGTTATGGTAATTGTGTGCCAGCMGCCGCGGTAA
v4.SA505	AATGATACGGCGACCACCGAGATCTACACTCATCGAGTATGGTAATTGTGTGCCAGCMGCCGCGGTAA
v4.SA506	AATGATACGGCGACCACCGAGATCTACACCGTGAGTGTATGGTAATTGTGTGCCAGCMGCCGCGGTAA
v4.SA507	AATGATACGGCGACCACCGAGATCTACACGGATATCTTATGGTAATTGTGTGCCAGCMGCCGCGGTAA
v4.SA508	AATGATACGGCGACCACCGAGATCTACACGACACCGTTATGGTAATTGTGTGCCAGCMGCCGCGGTAA
v4.SB501	AATGATACGGCGACCACCGAGATCTACACCTACTATATATGGTAATTGTGTGCCAGCMGCCGCGGTAA
v4.SB502	AATGATACGGCGACCACCGAGATCTACACCGTTACTATATGGTAATTGTGTGCCAGCMGCCGCGGTAA
v4.SB503	AATGATACGGCGACCACCGAGATCTACACAGAGTCACTATGGTAATTGTGTGCCAGCMGCCGCGGTAA
v4.SB504	AATGATACGGCGACCACCGAGATCTACACTACGAGACTATGGTAATTGTGTGCCAGCMGCCGCGGTAA
v4.SB505	AATGATACGGCGACCACCGAGATCTACACACGTCTCGTATGGTAATTGTGTGCCAGCMGCCGCGGTAA
v4.SB506	AATGATACGGCGACCACCGAGATCTACACTCGACGAGTATGGTAATTGTGTGCCAGCMGCCGCGGTAA
v4.SB507	AATGATACGGCGACCACCGAGATCTACACGATCGTGTTATGGTAATTGTGTGCCAGCMGCCGCGGTAA
v4.SB508	AATGATACGGCGACCACCGAGATCTACACGTCAGATATATGGTAATTGTGTGCCAGCMGCCGCGGTAA
v4.SA701	CAAGCAGAAGACGGCATACGAGATAACTCTCGAGTCAGTCAGCCGGACTACHVGGGTWTCTAAT
v4.SA702	CAAGCAGAAGACGGCATACGAGATACTATGTCAGTCAGTCAGCCGGACTACHVGGGTWTCTAAT

Name	5′–3′ sequence
v4.SA703	CAAGCAGAAGACGGCATACGAGATAGTAGCGTAGTCAGTCAGCCGGACTACHVGGGTWTCTAAT
v4.SA704	CAAGCAGAAGACGGCATACGAGATCAGTGAGTAGTCAGTCAGCCGGACTACHVGGGTWTCTAAT
v4.SA705	CAAGCAGAAGACGGCATACGAGATCGTACTCAAGTCAGTCAGCCGGACTACHVGGGTWTCTAAT
v4.SA706	CAAGCAGAAGACGGCATACGAGATCTACGCAGAGTCAGTCAGCCGGACTACHVGGGTWTCTAAT
v4.SA707	CAAGCAGAAGACGGCATACGAGATGGAGACTAAGTCAGTCAGCCGGACTACHVGGGTWTCTAAT
v4.SA708	CAAGCAGAAGACGGCATACGAGATGTCGCTCGAGTCAGTCAGCCGGACTACHVGGGTWTCTAAT
v4.SA709	CAAGCAGAAGACGGCATACGAGATGTCGTAGTAGTCAGTCAGCCGGACTACHVGGGTWTCTAAT
v4.SA710	CAAGCAGAAGACGGCATACGAGATTAGCAGACAGTCAGTCAGCCGGACTACHVGGGTWTCTAAT
v4.SA711	CAAGCAGAAGACGGCATACGAGATTCATAGACAGTCAGTCAGCCGGACTACHVGGGTWTCTAAT
v4.SA712	CAAGCAGAAGACGGCATACGAGATTCGCTATAAGTCAGTCAGCCGGACTACHVGGGTWTCTAAT
v4.SB701	CAAGCAGAAGACGGCATACGAGATAAGTCGAGAGTCAGTCAGCCGGACTACHVGGGTWTCTAAT
v4.SB702	CAAGCAGAAGACGGCATACGAGATATACTTCGAGTCAGTCAGCCGGACTACHVGGGTWTCTAAT
v4.SB703	CAAGCAGAAGACGGCATACGAGATAGCTGCTAAGTCAGTCAGCCGGACTACHVGGGTWTCTAAT
v4.SB704	CAAGCAGAAGACGGCATACGAGATCATAGAGAAGTCAGTCAGCCGGACTACHVGGGTWTCTAAT
v4.SB705	CAAGCAGAAGACGGCATACGAGATCGTAGATCAGTCAGTCAGCCGGACTACHVGGGTWTCTAAT
v4.SB706	CAAGCAGAAGACGGCATACGAGATCTCGTTACAGTCAGTCAGCCGGACTACHVGGGTWTCTAAT
v4.SB707	CAAGCAGAAGACGGCATACGAGATGCGCACGTAGTCAGTCAGCCGGACTACHVGGGTWTCTAAT
v4.SB708	CAAGCAGAAGACGGCATACGAGATGGTACTATAGTCAGTCAGCCGGACTACHVGGGTWTCTAAT
v4.SB709	CAAGCAGAAGACGGCATACGAGATGTATACGCAGTCAGTCAGCCGGACTACHVGGGTWTCTAAT
v4.SB710	CAAGCAGAAGACGGCATACGAGATTACGAGCAAGTCAGTCAGCCGGACTACHVGGGTWTCTAAT

Name	5′–3′ sequence
v4.SB711	CAAGCAGAAGACGGCATACGAGATTCAGCGTTAGTCAGTCAGCCGGACTACHVGGGTWTCTAAT
v4.SB712	CAAGCAGAAGACGGCATACGAGATTCGCTACGAGTCAGTCAGCCGGACTACHVGGGTWTCTAAT
Read 1 primer for V4 region	TATGGTAATTGTGTGCCAGCMGCCGCGGTAA
Read 2 primer for V4 region	AGTCAGTCAGCCGGACTACHVGGGTWTCTAAT
Index primer for V4 region	ATTAGAWACCCBDGTAGTCCGGCTGACTGACT
